# In Vitro Anticancer and Proapoptotic Activities of Steroidal Glycosides from the Starfish *Anthenea aspera*

**DOI:** 10.3390/md16110420

**Published:** 2018-11-01

**Authors:** Timofey V. Malyarenko, Olesya S. Malyarenko, Alla A. Kicha, Natalia V. Ivanchina, Anatoly I. Kalinovsky, Pavel S. Dmitrenok, Svetlana P. Ermakova, Valentin A. Stonik

**Affiliations:** 1 G.B. Elyakov Pacific Institute of Bioorganic Chemistry, Far Eastern Branch of the Russian Academy of Sciences, Pr. 100-let Vladivostoku 159, 690022 Vladivostok, Russia; malyarenko.os@gmail.com (O.S.M.); kicha@piboc.dvo.ru (A.A.K.); ivanchina@piboc.dvo.ru (N.V.I.); kaaniv@piboc.dvo.ru (A.I.K.); paveldmt@piboc.dvo.ru (P.S.D.); swetlana_e@mail.ru (S.P.E.); stonik@piboc.dvo.ru (V.A.S.); 2 Far Eastern Federal University, Sukhanova Str. 8, 690000 Vladivostok, Russia

**Keywords:** starfish, *Anthenea aspera*, steroidal glycosides, colony formation, apoptosis, Bcl-2 proteins

## Abstract

New marine glycoconjugates—the steroidal glycosides designated as anthenosides V–X (**1**–**3**)—and the seven previously known anthenosides E (**4**), G (**5**), J (**6**), K (**7**), S1 (**8**), S4 (**9**), and S6 (**10**) were isolated from the extract of the tropical starfish *Anthenea aspera*. The structures of **1**–**3** were elucidated by extensive NMR and ESIMS techniques. Glycoside **1** contains a rare 5α-cholest-8(14)-ene-3α,7β,16α-hydroxysteroidal nucleus. Compounds **2** and **3** were isolated as inseparable mixtures of epimers. All investigated compounds (**1**–**10**) at nontoxic concentrations inhibited colony formation of human melanoma RPMI-7951, breast cancer T-47D, and colorectal carcinoma HT-29 cells to a variable degree. The mixture of **6** and **7** possessed significant anticancer activity and induced apoptosis of HT-29 cells. The molecular mechanism of the proapoptotic action of this mixture was shown to be associated with the regulation of anti- and proapoptotic protein expression followed by the activation of initiator and effector caspases.

## 1. Introduction

Over the last decades, oncological diseases have become the second leading cause of death in the world, after cardiovascular diseases [1]. Nowadays, there are many therapeutic strategies applied to cancer treatment, including chemotherapy, surgery, radio- and immunotherapy, gene therapy, and target therapy. The last one is a type of cancer treatment that targets the changes in cancer cells to inhibit their growth, proliferation, and invasion [2]. Cancer cells are proved to be able to proliferate uncontrollably and avoid programmed cell death, apoptosis.

Apoptosis is a general biological process responsible for maintaining the constancy of the number of cells in cellular populations, as well as regulating the formation and elimination of defective cells. The main mediators of the intrinsic (mitochondrial) pathway of apoptosis are proteins of the Bcl-2 family, which are represented by antiapoptotic (Bcl-xL, Bcl-W, Mcl-1) and proapoptotic (Bax, Bak, Bad, Bim) proteins. They are considered to promote the alterations in mitochondrial membrane permeability required for the release of cytochrome c and other apoptogenic proteins that lead to the activation of caspases and the induction of apoptosis [3,4]. The search for and development of effective marine natural compounds that induce the apoptosis of cancer cells by targeting proteins of Bcl-2 family is a prospective strategy to control and stop cancer growth.

Some marine natural compounds have already been shown to possess significant anticancer activity in vitro and in vivo with relatively low toxicity, so marine organisms remain a highly productive source of promising cancer preventive or anticancer therapeutic agents [5]. It would be of particular interest to find natural compounds with relatively low cytotoxicity, but good cancer preventive properties: for example, the effective inhibition of microcolony formation of tumor cells at noncytotoxic doses.

Starfishes are known to contain diverse types of secondary metabolites possessing a wide spectrum of biological activities, including cytotoxic, antibacterial, neuritogenic, antifungal, cancer preventive, anti-inflammatory, and other effects [6,7,8,9,10,11,12]. At the same time, steroidal glycosides (glycosides of polyhydroxysteroids, cyclic glycosides, and asterosaponins) are common secondary metabolites of starfish. In most cases, polyhydroxysteroidal glycosides from starfish contain polyoxygenated steroidal aglycons (usually having from four to nine hydroxyl groups) and pentose (β-d-xylopyranosyl or α-l-arabinofuranosyl) or hexose (β-d-glucopyranosyl or β-d-galactofuranosyl) monosaccharide residues.

A series of glycosides of polyhydroxysteroids, named anthenosides, were previously isolated from starfishes of the genus *Anthenea* [13,14,15,16,17]. These compounds have a range of unusual structural features: Δ^8(14)^-3β,4β,6β,7β,16α-pentahydroxy, Δ^8(14)^-3β(α),6β,7β,16α-tetrahydroxy, or Δ^8(14)^-3α,7β,16α-trihydroxy steroidal nuclei; unoxidized side chains; and β-d-galactofuranosyl, 6-*O*-methyl-β-d-galactofuranosyl, 3-*O*-methyl-β-d-galactofuranosyl, 3-*O*-methyl-β-d-glucopyranosyl, 4-*O*-methyl-β-d-glucopyranosyl, or 2-acetamido-2-deoxy-4-*O*-methyl-β-d-glucopyranosyl residues.

It was previously reported that polyhydroxysteroidal glycosides from the starfish *Anthenea chinensis* exhibited significant activity against the promotion of tubulin polymerization in vitro, inhibiting the proliferation of human leukemia K-562, hepatoma BEL-7402, and spongioblastoma U87MG cell lines [14]. Recently, we demonstrated that some anthenosides from *Anthenea sibogae* slightly inhibited the proliferation and decreased the colony size of human breast cancer T-47D cells [16], whereas the anthenosides A_1_ and A_2_ from *A. aspera* slightly inhibited the cell viability of human cancer T-47D cells and did not show cytotoxic effects against human melanoma RPMI-7951 cells [17].

A few studies devoted to the proapoptotic activity of steroidal glycosides from starfishes have been reported. These compounds were proved to be able to induce mitochondrial apoptosis in glioblastoma U87MG cells [18], cause growth inhibition of human lung cancer A549 cells through the regulation of endoplasmic reticulum-induced apoptosis (ER-apoptosis) [19], and induce p53-dependent apoptosis by inhibition of AP-1, NF-κB, and ERK activities in human leukemia HL-60 and THP-1 cells [20]. These data confirm that polar steroids from starfishes are prospective anticancer compounds with proapoptotic activity. The aim of this work is to describe the isolation and structures of three new minor anthenosides V–X (**1**–**3**) and investigate the effects of these glycosides, as well as of a series of earlier known anthenosides isolated from *A. aspera*, on cell viability, colony formation, and induction of apoptosis in human melanoma RPMI-7951 as well as breast adenocarcinoma T-47D and colorectal carcinoma HT-29 cells.

## 2. Results and Discussion

**Figure 1:**
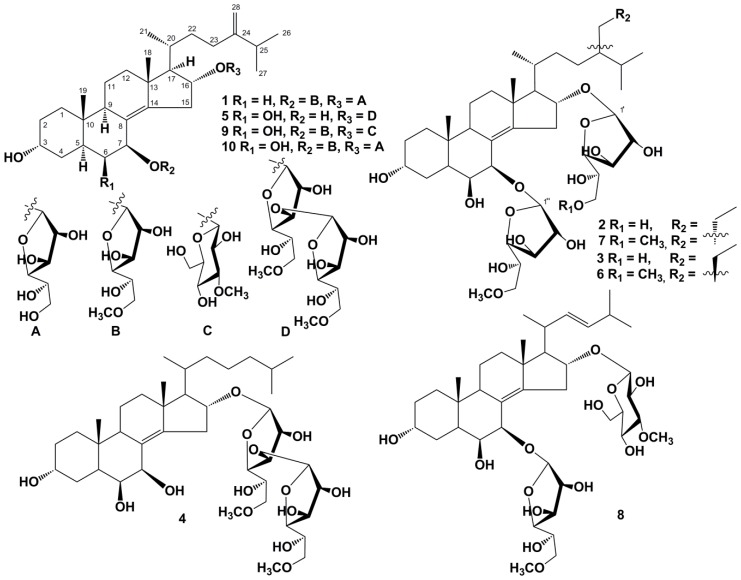
The structures of new compounds **1**–**10** isolated from *Anthenea aspera*.

The concentrated ethanol extract of *A. aspera* was subjected to sequential separation by chromatography on columns with Polychrom-1 and silica gel, followed by HPLC on semipreparative Diasorb-130-C16T and analytical Discovery C_18_ and Diasorb-130 Si gel columns to yield three new steroidal biglycosides (**1**−**3**), named anthenosides V–X (Figure 1), and seven previously known steroidal glycosides. The known compounds were identified by comparison of their ^1^H- and ^13^C-NMR and MS spectra with those reported for anthenoside E (**4**), anthenoside G (**5**), and the epimer mixture of the anthenosides J and K (**6** and **7**, ratio of 3:1, respectively) from *A. chinensis* [14], and anthenoside S1 (**8**), anthenoside S4 (**9**), and anthenoside S6 (**10**) from *A. sibogae* [16].

**Figure 2:**
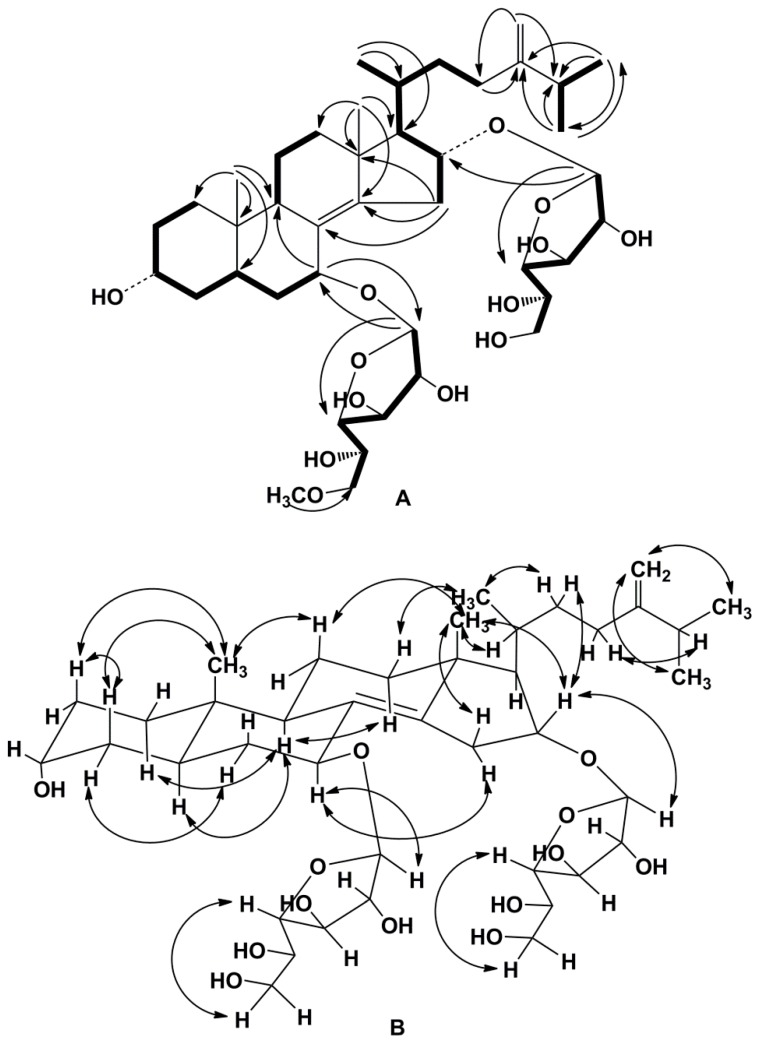
(**A**) ^1^H-^1^H COSY and key HMBC correlations for compound **1**. (**B**) Key ROESY correlations for compound **1**.

The molecular formula of compound **1** was determined to be of C_41_H_68_O_13_ from the [M + Na]^+^ sodiated adduct ion peak at *m*/*z* 791.4550 in the (+)HRESIMS spectrum (Figure S1). The ^1^H- and ^13^C-NMR spectral data belonging to the tetracyclic moiety of the aglycon of **1** showed the resonances of protons and carbons of two angular methyl groups, CH_3_-18 and CH_3_-19 (*δ*_H_ 0.90 s, 0.67 s; *δ*_C_ 20.2, 11.6), an 8(14) double bond (*δ*_C_ 128.9, 144.9), one oxygenated methine HC-3 (*δ*_H_ 3.98 t (*J* = 2.6); *δ*_C_ 67.1), and two oxygenated methine groups bearing the monosaccharide residues HC-7 (*δ*_H_ 4.40 t (*J* = 2.8); *δ*_C_ 74.0) and HC-16 (*δ*_H_ 4.43 td (*J* = 8.7, 4.6); *δ*_C_ 78.2) (Table 1, Figures S2 and S3). The ^1^H-^1^H COSY and HSQC correlations attributable to a steroidal nucleus revealed the corresponding sequences of protons from C-1 to C-7, C-9 to C-12 through C-11, and C-15 to C-17 (Figure 2A, Figures S4 and S5). Key HMBC cross-peaks, such as H-7/C-9; H-15/C-8, C-13, C-14; H_3_-18/C-12, C-13, C-14, C-17; and H_3_-19/С-1, С-5, С-9, С-10, confirmed the overall structure of the steroidal moiety of **1** (Figure 2A, Figure S6). The ROESY cross-peaks showed the common 5α/9α/10β/13β stereochemistry of the steroidal nucleus and 7β,16α-configurations of oxygenated substituents in **1** (Figure 2B, Figure S7). 

The NMR data attributable to the side chain of **1** indicated the existence of three secondary methyls, CH_3_-21 (*δ*_H_ 1.05 d (*J* = 6.6); *δ*_C_ 21.4), CH_3_-26 (*δ*_H_ 1.04 d (*J* = 6.7); *δ*_C_ 22.5), and CH_3_-27 (*δ*_H_ 1.04 d (*J* = 6.7); *δ*_C_ 22.3), and a 24(28) double bond (*δ*_H_ 4.76 brs, 4.72 brd (*J* = 1.3); *δ*_C_ 157.7, 107.1) (Table 1, Figures S2 and S3). The proton sequences from H-17 to H_3_-21 and H-23 and from H_3_-26 to H_3_-27 through H-25, correlating with the corresponding carbon atoms of the side chain of **1**, were assigned using the ^1^H-^1^H COSY and HSQC experiments (Figure 2A, Figures S4 and S5). The HMBC correlations H_3_-21/C-17, C-20; H-23/C-24; H_3_-26/C-24, C-25, C-27; H_3_-27/C-24, C-25, C-26; and H_2_-28/C-23, C-25 and the ROESY correlations H_3_-21/H-22; H-23/H-25; H_2_-28/H_3_-26, H_3_-27 supported the total structure of the Δ^24(28)^-24-methyl-cholestane side chain (Figure 2A,B, Figures S6 and S7) previously found in anthenosides F, G, and S4 [13,14]. A 20*R*-configuration was assumed on the basis of ROESY correlations of H_3_-18/H-20, H_3_-21; H_β_-16/H-22, and H_3_-21/H_β_-12. On the basis of all the above-mentioned data, the structure of the steroidal aglycon of **1** was determined to be (20*R*)-24-methyl-5α-cholesta-8(14),24(28)-diene-3α,7β,16α-triol.

The ^1^H-NMR spectrum of **1** showed two resonances at *δ*_H_ 4.94 and 4.99 in the deshielded region belonging to anomeric protons of the monosaccharide units that correlated in the HSQC spectrum with carbon atom signals at *δ*_C_ 107.6 and 107.3, respectively, as well as one resonance due to the *O*-methyl protons of the monosaccharide unit at *δ*_H_ 3.38, which correlated in the HSQC experiment with a carbon signal at *δ*_C_ 59.3 (Table 1, Figures S2 and S3). The fragment ion peaks at *m*/*z* 597 [(M + Na) − C_7_H_14_O_6_]^+^, 217 [С_7_H_14_O_6_ + Na]^+^, and 203 [С_6_H_12_O_6_ + Na]^+^ in the (+)ESIMS/MS spectrum from the precursor ion at *m*/*z* 791 [M + Na]^+^ showed the presence of *O*-methylhexose and hexose units in **1**. The chemical shifts and coupling constants of H-1−H-6 of the *O*-methylhexose and hexose units were determined by the irradiation of the anomeric proton atoms in the 1D TOCSY experiment. The application of ^1^H-^1^H COSY, HSQC, HMBC, and ROESY experiments led to the assignment of all carbon and proton resonances of the carbohydrate moieties in the NMR spectra of **1** (Table 1, Figure 2A,B). The carbon and proton signals and the corresponding coupling constants of the monosaccharide units agreed well with those of the terminal 6-*O*-methyl-β-galactofuranosyl residue and a β-galactofuranosyl residue in the NMR spectra of the mixture of anthenosides T and U [15]. The D-series of both monosaccharide units were expected by analogy with co-occurring anthenosides M and Q [15]. The attachments of the 6-*O*-methyl-β-d-galactofuranosyl and β-d-galactofuranosyl units to the steroidal aglycon were determined by the HMBC and ROESY spectra, in which the HMBC cross-peaks between H-1′ of Gal*_f_* and C-16 of the aglycon, H-1″ of 6-OMe-Gal*_f_* and C-7 of the aglycon were observed, and the ROESY cross-peaks between H-1′ of Gal*_f_* and H-16 of the aglycon and H-1″ of 6-OMe-Gal*_f_* and H-7 of the aglycon were observed. All these data allowed for the establishment of the structure of anthenoside V (**1**) as (20*R*)-7-*O*-(6-*O*-methyl-β-d-galactofuranosyl)-16-*O*-(β-d-galactofuranosyl)-24-methyl-5α-cholesta-8(14),24(28)-diene-3α,7β,16α-triol.

Compounds **2** and **3** were not separated by repeated reversed-phase HPLC. They have the same molecular formula, C_42_H_72_O_14_, determined from the [M + Na]^+^ sodiated adduct ion peak at *m*/*z* 823.4811 in the (+)HRESIMS spectrum (Figure S8). The ^1^H- and ^13^C-NMR spectroscopic data referring to the steroidal nucleus of the mixture of **2** and **3** revealed the chemical shifts of proton and carbon atoms of two angular methyl groups CH_3_-18 (*δ*_H_ 0.93 s, *δ*_C_ 20.2) and CH_3_-19 (*δ*_H_ 0.85 s, *δ*_C_ 15.4), an 8(14) double bond (*δ*_C_ 127.0, 147.3), two oxygenated methines HC-3 (*δ*_H_ 4.08 m; *δ*_C_ 67.5) and HC-6 (*δ*_H_ 3.62 t (*J* = 2.7); *δ*_C_ 75.2), as well as two oxygenated methines HC-7 (*δ*_H_ 4.22 d (*J* = 2.7); *δ*_C_ 78.3) and HC-16 (*δ*_H_ 4.49 td (*J* = 9.1, 5.5); *δ*_C_ 77.3) bearing *O*-monosaccharide residues (Table 1, Figures S9 and S10). The proton and carbon atom resonances of CH_3_-18, CH_3_-19, HC-3, HC-6, HC-7, and HC-16 were similar to the corresponding signals in the NMR spectra of most of the anthenosides from *A. chinensis* [14], *A. aspera* [15], and *A. sibogae* [16] and testified to a Δ^8(14)^-3α,6β,7β,16α-tetrahydroxysteroidal nucleus glycosylated at the C-7 and C-16 positions in **2** and **3** (Figures S11–S14). The NMR data of the side chains indicated the existence of three secondary methyls, CH_3_-21 (*δ*_H_ 1.05 d (*J* = 6.9); *δ*_C_ 21.7, 21.6), CH_3_-26 (*δ*_H_ 0.86 d (*J* = 6.8); *δ*_C_ 20.1), and CH_3_-27 (*δ*_H_ 0.85 d (*J* = 6.8); *δ*_C_ 19.3, 19.4), and one primary CH_3_-29 (*δ*_H_ 0.89 t (*J* = 7.4); *δ*_C_ 12.7, 12.4) (Table 1, Figures S9 and S10). The complexity of the methyl region of the ^13^C NMR data clearly showed the presence of two epimeric glycosides with 24*R* and 24*S* configurations. The proton and carbon resonances of the side chains, established by 2D NMR experiments, were similar to those of the side chains in the previously known anthenosides T and U [15] (Figures S11–S14). The chemical shift of C-29 in an ethyl group of the 24*S* epimer was more deshielded than that in the 24*R* epimer, while the chemical shifts of C-25, C-27, and C-28 of the 24*S* epimer were more shielded than those in the 24*R* epimer (Table 1, Figures S9 and S10). Therefore, the structures of the aglycon moieties of **2** and **3** were defined as (20*R*,24*S*)- and (20*R*,24*R*)-24-ethyl-5α-cholest-8(14)-ene-3α,6β,7β,16α-tetraols, respectively. Evaluation of the intensities of the C-29 resonances revealed a ratio of 3:1 for the **2**:**3** in the mixture, showing the predominance of the 24*S* epimer (**2**).

The ^1^H-NMR spectrum of the mixture of **2** and **3** showed two resonances at *δ*_H_ 4.96 and 4.99 in the deshielded region belonging to anomeric protons of the monosaccharide units that correlated in the HSQC spectrum with carbon atom signals at *δ*_C_ 107.7 and 108.4, respectively, as well as one resonance due to *O*-methyl protons of the monosaccharide unit at *δ*_H_ 3.39, correlated in the HSQC experiment with a carbon signal at *δ*_C_ 59.3 (Table 1, Figures S9 and S10). A detailed comparison of the ^1^H- and ^13^C-NMR data of monosaccharide units with those of **1** clearly indicated that compounds **2** and **3** contained the same 6-*O*-methyl-β-galactofuranosyl and β-galactofuranosyl residues (Table 1). The attachments of the 6-*O*-methyl-β-d-galactofuranosyl and β-d-galactofuranosyl units to the steroidal aglycon were determined by the HMBC and ROESY spectra, where the HMBC cross-peaks between H-1′ of Gal*_f_* and C-16 of the aglycon, H-1″ of 6-OMe-Gal*_f_* and C-7 of the aglycon, as well as the ROESY cross-peaks between H-1′ of Gal*_f_* and H-16 of the aglycon and H-1″ of 6-OMe-Gal*_f_* and H-7 of the aglycon, were observed. Therefore, the structures of anthenosides W (**2**) and X (**3**) were determined to be (20*R*,24*S*)- and (20*R*,24*R*)-7-*O*-(6-*O*-methyl-β-d-galactofuranosyl)-16-*O*-(β-d-galactofuranosyl)-24-ethyl-5α-cholest-8(14)-ene-3α,6β,7β,16α-tetraols, respectively.

**The cytotoxic activity of compounds 1–10 from *A. aspera*.** The study on the biological activities of the tested compounds involved the determination of their cytotoxicity as the first step. The MTS method was used to assess the cytotoxic activity of glycosides **1**–**10** against the human melanoma RPMI-7951, breast cancer T-47D, and colorectal carcinoma HT-29 cell lines. Cells were treated with compounds **1**–**10** in concentrations ranging from 20 to 160 μM and incubated for 24 h, as described in the section titled “Experimental”.

All investigated compounds, except the mixture of **6** and **7**, did not cause inhibition of the cell viability of RPMI-7951, T-47D, and HT-29 cells at concentrations up to 160 μM. The mixture of **6** and **7** possessed a comparable cytotoxic effect against RPMI-7951, T-47D, and HT-29 cell lines with IC_50_ values of 89, 91, and 85 μM, respectively.

**The inhibitory activity of glycosides 1–10 against colony formation of human cancer cells.** In the last decade, the influence of polar steroids from starfishes on the formation and growth of colonies of various types of cancer cells has been intensively studied [21,22,23].

**Figure 3:**
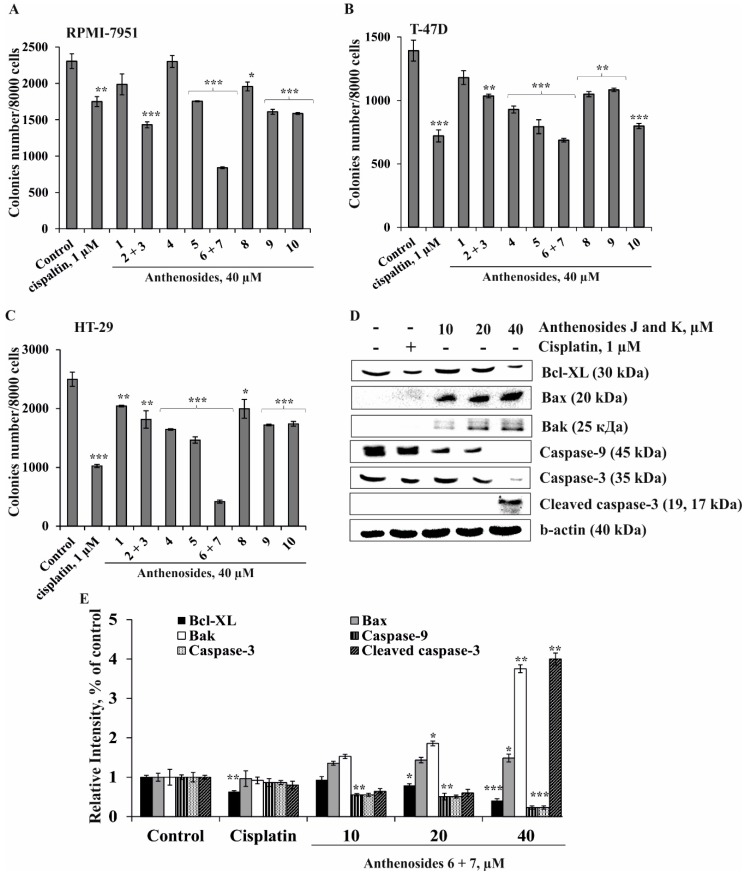
The effects of anthenosides **1**–**10** on colony formation of human melanoma, breast cancer, and colorectal carcinoma cells and on induction of apoptosis of HT-29 cells. RPMI-7951 (**A**), T-47D (**B**), and HT-29 (**C**) cells (2.4 × 10^4^/mL) treated with/without the investigated compounds (40 µM) or cisplatin (1 µM) (positive control) were exposed to 1 mL of 0.3% Basal Medium Eagle (BME) agar containing 10% FBS and overlaid with 3.5 mL of 0.5% BME agar containing 10% FBS. The culture was maintained at 37 °C in a 5% CO_2_ atmosphere for 2 weeks. The colonies were counted under a microscope with the aid of the ImageJ software program. The significant differences were evaluated using Student’s *t* test. The asterisks indicate a significant decrease in colony formation of cancer cells treated with the tested compounds or cisplatin compared to the non-treated cells (control), * *p* < 0.05, ** *p* < 0.01, *** *p* < 0.001. (**D**) Regulation of anti- and proapoptotic protein expression, as well as initiator and effector caspase activity, by the mixture of anthenosides J (**6**) and K (**7**) in HT-29 cells. HT-29 cells were either treated by cisplatin (1 µM) or the mixture of **6** + **7** (10, 20, and 40 µM) for 24 h. After drug treatment, total protein lysates were prepared. The protein samples (30 µg) were subjected to SDS-PAGE, followed by detection with immunoblotting using antibodies against Bcl-XL (30 kDa), Bax (20 kDa), Bak (25 kDa), caspase-9 (45 kDa) and -3 (35 kDa), cleaved caspase-3 (17, 19 kDa), and b-actin (40 kDa) proteins. (**E**) Relative band intensity was measured using Image Lab™ Software (“Bio Rad”, Hercules, CA, USA). The quantitative results are presented as the mean value from three independent experiments. The significant differences were evaluated using Student’s *t* test. The asterisks indicate a significant alteration of the proteins’ expression in cells treated by cisplatin or the mixture of **6** + **7** compared with the non-treated cells (control), * *p* < 0.05, ** *p* < 0.01, *** *p* < 0.001.

In the present work, the effects of compounds **1**–**10** on the formation and growth of colonies of human melanoma, breast cancer, and colorectal carcinoma were determined using the soft agar method, which is considered to be the most accurate type of in vitro assay for detecting the malignant transformation of cells [24]. Compounds **1**–**10** at a noncytotoxic concentration of 40 µM were shown to inhibit spontaneous colony formation and growth of RPMI-7951, T-47D, and HT-29 cells to a variable degree (Figure 3A–C). All compounds, except the mixture of **6** + **7**, inhibited colony formation of the investigated cells by less than 30%, compared to non-treated cells (control). Anthenosides J and K possessed significant inhibitory activity against all tested cell lines; these glycosides decreased the number of colonies of RPMI-7951, T-47D, and HT-29 cancer cells by 64%, 55%, and 83%, respectively, compared to non-treated cells (control). The chemotherapeutic drug cisplatin, used as a positive control in this study, at a noncytotoxic dose (1 µM) inhibited colony formation of RPMI-7951, T-47D, and HT-29 cells by 24%, 48%, and 59%, respectively, compared to controls (Figure 3A–C).

Compounds **4**, **5**, and **10** (40 µM) effectively inhibited colony formation of HT-29 and T-47D cells to a comparable degree by 34% and 33%; 41% and 43%; and 30% and 41%, respectively. Compounds **1**, **2** + **3**, **8**, and **9** at the same concentration had a moderate inhibitory activity and suppressed colony formation of melanoma, breast cancer, and colorectal adenocarcinoma cells by less than 30%. The colorectal carcinoma cells HT-29 were found to be the most sensitive to the effect of the anthenosides, while the melanoma cells RPMI-7951 were the most chemoresistant. Recently, similar effects of sulfated polar steroids from the Far Eastern starfish *Leptasterias ochotensis*, as well as monoglycosides, anthenosides A1 and A2, and anthenoside A from the starfish *Anthenea aspera*, were reported. These compounds were able to slightly inhibit colony formation of melanoma cell lines and effectively decrease the number and size of colonies of breast and colorectal cancer cells [17,25]. We suggest that the further search for cancer preventive agents in the form of natural products from starfish may lead to more active compounds. 

It is likely that the effective inhibitory activity of the investigated compounds against colorectal carcinoma cells can be explained by the regulation of specific molecules involved in a signaling cascade which is activated in these cancer cells. Since the mixture of anthenosides J (**6**) and K (**7**) exerted the greatest anticancer activity against the colorectal carcinoma cells HT-29, they were chosen for further investigation of their proapoptotic activity in this cell line.

**The proapoptotic activity of the mixture of anthenosides J (6) and K (7) in human colon carcinoma cells.** The induction of apoptosis of cancer cells is known to be one of the accepted strategies of modern chemotherapy. In the present study, we supposed that the significant inhibitory activity of colony formation observed for colon carcinoma cells by the mixture of anthenosides J (**6**) and K (**7**) was due to the induction of apoptosis.

Apoptosis is a multistage process and can be realized by several signaling pathways. The mechanism of cell death mediated through the activation of cell death receptors on the cell surface is the extrinsic pathway of apoptosis, while the signaling cascade initiated through mitochondria is named the intrinsic pathway of apoptosis [26].

Among the major regulators of the intrinsic pathway are the caspases and Bcl-2 protein family members. Caspases are cysteine aspartate-specific protein kinases. Based on their function, caspases are divided into two groups: the initiator and the effector caspases. The initiator caspases (caspase-2, -8, -9, and -10) activate the effector caspases, while the effector caspases (caspase-6, -7, and -3) cause the degradation of specific substrates, disrupting the integration of cellular subsystems [4].

It is known that the Bcl-2 protein family controls the membrane permeability of mitochondria and is represented by the proapoptotic BH3-only proteins (Bid, Bim, Puma, Noxa, Bad, Bmf, Hrk, and Bik); the prosurvival Bcl-2-like proteins (Bcl-2, Bcl-XL, Bcl-XS, Bcl-w, BAG); and the pore-forming Bax and Bak proteins [27]. The BH3-only proteins may directly bind and activate Bax and Bak, and also bind to the prosurvival Bcl-2-like proteins to indirectly activate Bax and Bak. Once activated, Bax and Bak oligomerize to form pores in the mitochondrial outer membrane that release cytochrome c. Cytosolic cytochrome c leads to caspase activation and subsequent cell death. These proteins have special significance since they can determine whether the cell commits to apoptosis or aborts the process [28].

In the present study, the capability of the mixture of anthenosides J (**6**) and K (**7**) to regulate the expression of proteins in the Bcl-2 family, as well as activate the initiator or effector caspases, was first determined.

Anthenosides J (**6**) and K (**7**) were shown to downregulate the expression of the antiapoptotic Bcl-XL protein and upregulate the expression of the proapoptotic proteins Bax and Bak that lead to the activation of the initiator caspase-9. Activated caspase-9, in turn, induced the upregulation of the effector caspase-3 expression in a dose-dependent manner. As a result, the mixture of the investigated compounds **6** + **7** at 40 µM caused proteolytic cleavage of caspase-3 and led to apoptosis of colorectal carcinoma cells (Figure 3D). Recently, we demonstrated that luzonicosides from the starfish *Echinaster luzonicus* induced the apoptosis of human melanoma cells by the regulation of p21, cyclin D1, caspase-3, Bcl-2, and Survivin protein expression levels [29]. It was also shown that novaeguinoside II from the starfish *Culcita novaeguineae* induced apoptosis in human glioblastoma U87MG cells by increasing cytochrome-c release from mitochondria, depolarizing of ΔΨm, and by activating caspase 3, followed by DNA degradation [18]. Asterosaponin 1 was found to cause the inhibition of proliferation of human lung cancer A549 cells through the regulation of endoplasmic reticulum-induced apoptosis (ER-apoptosis) [19]. Leviusculoside G from the starfish *Henricia leviuscula* induced p53-dependent apoptosis by inhibition of AP-1, NF-κB, and ERK activities in human leukemia HL-60 and THP-1 cells [20].

It is interesting to note that plancitoxin I, the major lethal factor from the crown-of-thorns starfish *Acanthaster planci* venom, also possesses strong proapoptotic activity that is realized by a caspase 3-independent apoptotic pathway in rat liver TRL 1215 cells [30]. Moreover, this toxin was found to increase the reactive oxygen species (ROS) formation, which induces mitochondrial depolarization, and the elevation of p38 expression; it then can induce the fragmentation of nuclear DNA, triggering apoptosis [31].

In conclusion, our data provide evidence that the mixture of **6** + **7** significantly inhibited colony formation of human melanoma, breast cancer, and colorectal carcinoma cells and induced apoptosis in colorectal carcinoma HT-29 cells through the regulation of anti- and proapoptotic protein expression and the activation of initiator and effector caspases. 

## 3. Materials and Methods

### 3.1. General Procedures

Optical rotations were determined on a PerkinElmer 343 polarimeter (Waltham, MA, USA). The ^1^H- and ^13^C-NMR spectra were recorded on a Bruker Avance III 700 spectrometer (Bruker, Germany) at 700.13 and 176.04 MHz, respectively, and chemical shifts were referenced to the corresponding residual solvent signal (*δ*_H_ 3.30/*δ*_C_ 49.0 for CD_3_OD). The HRESIMS spectra were recorded on a Bruker Impact II Q-TOF mass spectrometer (Bruker, Germany); the samples were dissolved in MeOH (*c* 0.001 mg/mL). HPLC separations were carried out on an Agilent 1100 Series chromatograph (Agilent Technologies, Santa Clara, CA, USA) equipped with a differential refractometer; Diasorb-130-C16T (11 µm, 250 × 16 mm, Biochemmack, Moscow, Russia), Discovery C_18_ (5 µm, 250 × 4 mm, Supelco, North Harrison, PA, USA), and Diasorb-130 Si gel columns (6 µm, 250 × 4.6 mm, Biochemmack, Moscow, Russia) were used. Low-pressure liquid column chromatography was carried out with Polychrom-1 (powdered Teflon, 0.25–0.50 mm; Biolar, Olaine, Latvia) and Si gel KSK (50–160 µm, Sorbpolimer, Krasnodar, Russia). Sorbfil Si gel plates (4.5 × 6.0 cm, 5–17 µm, Sorbpolimer, Krasnodar, Russia) were used for thin-layer chromatography.

### 3.2. Animal Material

Specimens of *Anthenea aspera* Döderlein, 1915 (order Valvatida, family Oreasteridae) were collected at a depth of 3–20 m by hand via scuba at Tu Long Bay near Khuan Lan Island in the South China (East) Sea during the research vessel Akademik Oparin’s 34th scientific cruise in May 2007. Species identification was carried out by Dr. T.I. Antokhina (Severtsov Institute of Ecology and Evolution, RAS, Moscow). A voucher specimen (no. 034-142) is on deposit at the marine specimen collection of the G.B. Elyakov Pacific Institute of Bioorganic Chemistry of the FEB RAS, Vladivostok, Russia.

### 3.3. Extraction and Isolation

The fresh specimens of *A. aspera* (1.3 kg, crude weight) were chopped into small pieces and extracted thrice with EtOH. The H_2_O/EtOH layer was evaporated, and the residue was dissolved in H_2_O (1.0 L). The H_2_O-soluble material was passed through a Polychrom-1 column (7 × 26.5 cm), eluted with distilled H_2_O (4.0 L) until a negative chloride ion reaction was obtained, and then eluted with EtOH (3.5 L). The combined EtOH eluate was evaporated to give a reddish residue (7.0 g). This material was chromatographed over a Si gel column (6 × 18.5 cm) using CHCl_3_/EtOH (stepwise gradient, 6:1–EtOH, *v*/*v*) to yield eight fractions, 1–8, which were then analyzed by TLC in the eluent system BuOH/EtOH/H_2_O (4:1:2, *v*/*v*/*v*). Fractions 1–6 mainly contained the polyhydroxysteroids and related glycosides and admixtures of pigments and concomitant lipids. HPLC separation of fractions 2 (113 mg) and 5 (212 mg) on a Diasorb-130-C16T column (2.5 mL/min) with EtOH/H_2_O (75:25, *v*/*v*) as an eluent system yielded pure **4** (7.5 mg, R_t_ 22.1 min) and **5** (3.5 mg, R_t_ 26.7 min) and subfractions 2.4 (4.5 mg) and 5.7 (13.0 mg), which were additionally submitted for purification on a Discovery C_18_ analytical column (1.0 mL/min) with EtOH/H_2_O (70:30, *v/v*) as an eluent system to give subfractions 2.42 (2.5 mg) and 5.72 (8.5 mg), respectively. HPLC separation of subfractions 2.42 and 5.72 on a Diasorb-130 Si gel analytical column (1.0 mL/min) with EtOAc/EtOH (30:1, *v*/*v*) as an eluent system yielded pure **1** (0.5 mg, R_t_ 24.3 min), a mixture of **2** and **3** (1.0 mg, R_t_ 54.7 min), and pure **8** (1.5 mg, R_t_ 28.5 min), **9** (2.8 mg, R_t_ 31.7 min), and **10** (3.5 mg, R_t_ 36.6 min). HPLC separation of fraction 4 (183 mg) on a Diasorb-130-C16T column (2.5 mL/min) with EtOH/H_2_O (70:30, *v*/*v*) as an eluent system yielded a mixture of **6** and **7** (9.3 mg, R_t_ 50.2 min).

### 3.4. Compound Characterization Data

*Anthenoside V* [(20*R*)-7-*O*-(6-*O*-methyl-β-d-galactofuranosyl)-16-*O*-(β-d-galactofuranosyl)-24-methyl-5α-cholest-8(14),24(28)-diene-3α,7β,16α-triol] (**1**): Amorphous powder; ${\left[\alpha \right]}_{25}^{D}$: –22.8 (*c* 0.05, MeOH); IR (CDCl_3_) ν_max_ 3418, 3021, 2856, 1603, 1260, 1216, 1034 cm^−1^; HRESIMS *m*/*z* 791.4550 [M + Na]^+^ (calcd. for C_41_H_68_O_13_, 791.4549); ESIMS/MS of the ion at *m*/*z* 791: 597 [(M + Na)–C_7_H_14_O_6_]^+^, 217 [С_7_H_14_O_6_ + Na]^+^, 203 [С_6_H_12_O_6_ + Na]^+^; ^1^H- and ^13^C-NMR data, see Table 1.

*Mixture of the anthenosides W and X* (20*R*,24*S*)- and (20*R*,24*R*)-7-*O*-(6-*O*-methyl-β-d-galactofuranosyl)-16-*O*-(β-d-galactofuranosyl)-24-ethyl-5α-cholest-8(14)-ene-3α,6β,7β,16α-tetraols (**2** + **3**, ratio of 3:1): Amorphous powder; ${\left[\alpha \right]}_{25}^{D}$: –33.1 (*c* 0.1, MeOH); IR (CDCl_3_) ν_max_ 3420, 3020, 2857, 1603, 1261, 1216, 1034 cm^−1^; HRESIMS *m*/*z* 823.4811 [M + Na]^+^ (calcd. for C_42_H_72_O_14_, 823.4814); ESIMS/MS of the ion at *m*/*z* 823: 629 [(M + Na)–C_7_H_14_O_6_]^+^, 217 [С_7_H_14_O_6_ + Na]^+^, 203 [С_6_H_12_O_6_ + Na]^+^; ESIMS/MS of the ion at *m*/*z* 799: 605 [(M − H)–C_7_H_14_O_6_]^–^, 443 [(M − H)–C_7_H_14_O_6_–C_6_H_12_O_6_]^−^; ^1^H- and ^13^C-NMR data, see Table 1.

### 3.5. Bioactivity Assay

#### 3.5.1. Reagents

Phosphate-buffered saline (PBS), l-glutamine, penicillin–streptomycin solution (10,000 U/mL, 10 µg/mL) were from the “Sigma-Aldrich” company (St. Louis, MO, USA).

MTS reagent—3-[4,5-dimethylthiazol-2-yl]-2,5-diphenyltetrazolium bromide—was purchased from “Promega” (Madison, WI, USA).

Basal Medium Eagle (BME), Dulbecco’s Modified Eagle’s Medium (DMEM), Minimum Essential Medium Eagle (MEM), McCoy’s 5A Modified Medium (McCoy’s 5A), trypsin, fetal bovine serum (FBS), agar, and the protein marker “PageRuler Plus Prestained Protein Ladder” were purchased from “ThermoFisher Scientific” (Waltham, MA, USA).

Primary antibodies against caspase-9, caspase-3, cleaved caspase-3, Bcl-XL, Bax, Bak, and b-actin, and horseradish peroxidase (HRP)-conjugated secondary antibody from rabbit and mouse were obtained from “Cell Signaling Technology” (Danvers, MA, USA).

#### 3.5.2. Cell Lines and Culture Conditions

Human malignant melanoma RPMI-7951 cells (ATCC^®^ no. HTB-66™), breast cancer T-47D cells (ATCC^®^ no. HTB-133™), and colorectal carcinoma HT-29 cells (ATCC^®^ no. HTB-38™) were obtained from the American Type Culture Collection (Manassas, WV, USA).

RPMI-7951, T-47D, and HT-29 cells were cultured in 200 µL of complete DMEM/10% FBS and RPMI-1640/10% FBS, and McCoy’s 5A medium, respectively, and 1% penicillin–streptomycin solution. The cell cultures were maintained at 37 °C in a humidified atmosphere containing 5% CO_2_. Every 3–4 days cells were rinsed with PBS, detached from the tissue culture flask by 0.25% trypsin/0.05 M EDTA, and 10–20% of the harvested cells were transferred to a new flask containing fresh culture media.

#### 3.5.3. Cell Viability Assay

RPMI-7951 (1.0 × 10^4^), T-47D (1.2 × 10^4^), and HT-29 (1.0 × 10^4^) cells were cultured in 200 µL of complete DMEM/10% FBS and RPMI-1640/10% FBS, and McCoy’s 5A medium, respectively, for 24 h at 37 °C in 5% CO_2_ incubator. The cell monolayer was treated with PBS (control), cisplatin (1 µM), and various concentrations of compounds **1**–**10** (20, 40, 80, and 160 µM) for 24 h. Subsequently, the cells were incubated with 15 µL MTS reagent for 3 h, and the absorbance of each well was measured at 490/630 nm using a Power Wave XS microplate reader (“BioTek”, Wynusky, VT, USA).

#### 3.5.4. The Soft Agar Colony Formation Assay

Cells (2.4 × 10^4^/mL) were seeded in a 6-well plate and treated with compounds (20 and 40 µM) in 1 mL of 0.3% Basal Medium Eagle (BME) agar containing 10% FBS, 2 mM l-glutamine, and 25 µg/mL gentamicin. The cultures were maintained at 37 °C in a 5% CO_2_ incubator for 14 days, and the cell colonies were scored using a microscope (Motic AE 20, XiangAn, Xiamen, China) and the ImageJ software.

#### 3.5.5. Western Blotting

HT-29 cells (6 × 10^5^) were seeded in a 10 cm dish overnight and cultured for 24 h. Then, they were treated with cisplatin (1 µM) or the mixture of compounds **6** and **7** (10, 20, and 40 µM) for 48 h. Then, the cells were harvested and centrifuged at 5000 rpm for 5 min. The harvested cells were lysed with lysis buffer (50 mM Tris–HCl (pH 7.4), 150 mM NaCl, 1 mM EDTA, 1 mM EGTA, 10 mg/mL aprotinin, 10 mg/mL leupeptin, 5 mM phenylmethanesulfonyluoride (PMSF), 1 mM dithiolthreitol (DTT) containing 1% Triton X-100) and centrifuged at 12,000 rpm for 15 min to removed insoluble debris. The protein content was determined using Bradford reagent (“Bio-Rad”, Hercules, CA, USA). Lysate protein (20–40 µg) was subjected to 12% SDS-PAGE and electrophoretically transferred to polyvinylidene difluoride membranes (PVDF) (“Millipore”, Burlington, MA, USA). The membranes were blocked with 5% non-fat milk for 1 h and then incubated with the respective specific primary antibody at 4 °C overnight. Protein bands were visualized using an enhanced chemiluminescence reagent (ECL) (“Bio Rad”, Hercules, CA, USA) after hybridization with an HRP-conjugated secondary antibody. Band density was quantified using the ImageJ software.

#### 3.5.6. Statistical Analysis

All assays were performed in at least three independent experiments. Results are expressed as the mean ± standard deviation (SD). Student’s *t* test was used to evaluate the data with the following significance levels: * *p* < 0.05, ** *p* < 0.01, *** *p* < 0.001. 

## 4. Conclusions

Three new minor steroidal glycosides—anthenosides V−X (**1**–**3**)—and the seven previously known anthenosides E (**4**), G (**5**), J (**6**), K (**7**), S1 (**8**), S4 (**9**), and S6 (**10**) were isolated from the extract of the tropical starfish *Anthenea aspera*. Anthenoside V (**1**) has the rare steroidal aglycon without the OH-group at C-6. Previously, only five steroidal glycosides with a non-oxygenated C-6 atom—anthenosides T and U from *A. aspera* and kurilensosides E, F, and G from *Hippasteria kurilensis* [32]—had been found in starfishes. Anthenosides W and X (**2** and **3**) were isolated as unseparated mixtures of epimers. Previously, glycoside pairs with the same side chains—anthenosides H and I, anthenosides J and K, and anthenosides T and U—were found in the starfishes *A. chinensis* and *A. aspera* [14,15] also as unseparated mixtures of epimers. Anthenoside **1**–**10** at noncytotoxic concentrations inhibited the formation and growth of colonies of human melanoma RPMI-7951, breast cancer T-47D, and colorectal carcinoma HT-29 cells to a variable degree. The mixture of the anthenosides **6** + **7** possessed the most significant inhibitory activity against all tested cancer cell lines among the investigated compounds. The molecular mechanism of anticancer activity of these compounds was associated with the induction of apoptosis of colorectal carcinoma HT-29 cells through the regulation of anti- and proapoptotic protein expression followed by the activation of initiator and effector caspases. Further investigations on the detailed molecular mechanism of the anticancer effect of anthenosides from starfishes are needed to provide new approaches to the development of effective cancer therapy regimens.

## Figures and Tables

**Table 1:** ^1^H- (700.13 MHz) and ^13^C-NMR (176.04 MHz) chemical shifts of **1** and the mixture of **2** and **3** in CD_3_OD at 30 °C; *δ* in ppm, *J* values in Hz.

Position		1			Mixture of 2 and 3	
	DEPT	*δ*_H_	*δ*_C_	DEPT	*δ*_H_	*δ*_C_
1β 1α	CH_2_	1.52 dd (12.3, 5.1) 1.39 m	32.8	CH_2_	1.53 m 1.30 m	34.5
2	CH_2_	1.62 m	29.6	CH_2_	1.62 m	29.9
3	CH	3.98 t (2.6)	67.1	CH	4.08 m	67.5
4β 4α	CH_2_	1.44 m 1.37 m	36.3	CH_2_	1.96 td (13.6, 2.7) 1.37 m	33.3
5	CH	2.17 m	33.1	CH	2.12 dt (13.6, 2.7)	38.0
6	CH_2_	1.55 dt (14.2, 2.8) 1.23 m	34.3	CH	3.62 t (2.7)	75.2
7	CH	4.40 t (2.8)	74.0	CH	4.22 d (2.7)	78.3
8	C		128.9	C		127.0
9	CH	2.25 m	46.3	CH	2.26 m	45.9
10	C		38.6	C		38.8
11β 11α	CH_2_	1.65 m 1.45 m	19.8	CH_2_	1.65 m 1.54 m	19.5
12β 12α	CH_2_	1.81 m 1.26 m	37.2	CH_2_	1.80 dt (12.5, 3.6) 1.24 m	37.1
13	C		44.8	C		45.0
14	C		144.9	C		147.3
15β 15α	CH_2_	2.77 ddd (16.8, 8.7, 3.0) 2.56 ddd (16.8, 4.6, 1.8)	33.7	CH_2_	2.88 ddd (17.1, 9.1, 3.2) 2.59 ddd (17.1, 5.5, 2.0)	33.7
16	CH	4.43 td (8.7, 4.6)	78.2	CH	4.49 td (9.1, 5.5)	77.3
17	CH	1.46 t (4.6)	62.9	CH	1.47 dd (9.1, 3.9)	63.0
18	CH_3_	0.90 s	20.2	CH_3_	0.93 s	20.2
19	CH_3_	0.67 s	11.6	CH_3_	0.85 s	15.4
20	CH	1.67 m	33.3	CH	1.60 m	33.5 33.4
21	CH_3_	1.05 d (6.6)	21.4	CH_3_	1.05 d (6.9)	21.7 21.6
22	CH_2_	1.81 m 1.43 m	33.9	CH_2_	1.65 m 1.26 m	33.2
23	CH_2_	2.23 m 1.95 m	33.7	CH_2_	1.47 m 1.09 m	29.7 29.4
24	C		157.7	CH	1.05 m	47.2 47.0
25	CH	2.26 m	35.0	CH	1.75 m	30.2 30.5
26	CH_3_	1.04 d (6.7)	22.5	CH_3_	0.86 d (6.8)	20.1
27	CH_3_	1.04 d (6.7)	22.3	CH_3_	0.85 d (6.8)	19.3 19.4
28	CH_2_	4.76 brs 4.72 brd (1.3)	107.1	CH_2_	1.37 m 1.20 m	24.0 24.2
29				CH_3_	0.89 t (7.4)	12.7 12.4
**β-d-Gal*_f_***						
1′	CH	4.94 brd (2.4)	107.6		4.96 brd (2.2)	107.7
2′	CH	3.95 m	83.6		3.96 dd (4.6, 2.2)	83.7
3′	CH	4.03 dd (7.0, 4.8)	78.3		4.04 dd (7.0, 4.6)	78.4
4′	CH	3.88 dd (7.0, 2.9)	84.4		3.89 dd (7.0, 3.0)	84.6
5′	CH	3.72 m	72.4		3.73 ddd (7.6, 4.5, 3.0)	72.5
6′	CH_2_	3.61 dd (11.3, 7.5) 3.58 dd (11.3, 4.8)	65.5		3.64 dd (11.2, 7.6) 3.61 dd (11.2, 4.5)	65.4
**6-OMe-β-d-Gal*_f_***						
1′	CH	4.99 brd (2.0)	107.3		4.99 brd (1.9)	108.4
2′	CH	3.93 dd (3.9, 2.0)	83.5		3.91 dd (3.9, 1.9)	83.4
3′	CH	3.95 m	78.8		3.95 dd (6.1, 3.9)	78.7
4′	CH	3.90 dd (6.1, 3.7)	84.8		3.87 dd (6.1, 3.6)	85.0
5′	CH	3.83 m	70.8		3.84 m	70.7
6′	CH_2_	3.53 dd (10.1, 4.6) 3.50 dd (10.1, 7.2)	75.4		3.53 d (6.0)	75.5
OCH_3_	CH_3_	3.38 s	59.3		3.39 s	59.3

## Supplementary Materials

The following are available online at http://www.mdpi.com/1660-3397/16/11/420/s1. Copies of HRESIMS (Figures S1 and S8), ^1^H-NMR (Figures S2 and S9), ^13^C-NMR (Figures S3 and S10), ^1^H-^1^H-COSY (Figures S4 and S11), HSQC (Figures S5 and S12), HMBC (Figures S6 and S13), and ROESY (Figures S7 and S14) spectra of compounds **1** and the mixture of **2** and **3**, respectively. This material is available free of charge online.
