# Supplementary material for: In Vitro Anticancer and Proapoptotic Activities of Steroidal Glycosides from the Starfish Anthenea aspera
Source: Mar Drugs. 2018 Nov 1;16(11):420. doi: 10.3390/md16110420 (PMC6266882; doi:10.3390/md16110420)

Supplementary Materials

In vitro Anticancer and Pro-apoptotic Activities of Steroidal Glycosides from the Starfish *Anthenea aspera*

Timofey V. Malyarenko[1,2*], Olesya S. Malyarenko[1], Alla A. Kicha[1], Natalia V. Ivanchina[1], Anatoly I. Kalinovsky[1], Pavel S. Dmitrenok[1], Svetlana P. Ermakova[1] and Valentin A. Stonik[1,2]

[1] G.B. Elyakov Pacific Institute of Bioorganic Chemistry, Far Eastern Branch of the Russian Academy of Sciences, Pr. 100-let Vladivostoku 159, 690022 Vladivostok, Russia
malyarenko.os@gmail.com (O.S.M.); kicha@piboc.dvo.ru (A.A.K.); ivanchina@piboc.dvo.ru (N.V.I.); kaaniv@piboc.dvo.ru (A.I.K.); paveldmt@piboc.dvo.ru (P.S.D.); swetlana_e@mail.ru (S.P.E.); stonik@piboc.dvo.ru (V.A.S.).

[2] Far Eastern Federal University, Sukhanova str. 8, 690000 Vladivostok, Russia

* Correspondence: malyarenko-tv@mail.ru (T.V.M.) Tel.: +7-423-2312-360; Fax: +7-423-2314-050.

List

Figure S1. HRESIMS spectrum of anthenoside V (**1**).

Figure S2. ^{1}H-NMR spectrum of anthenoside V (**1**) in CD_3OD.

Figure S3. ^{13}C-NMR spectrum of anthenoside V (**1**) in CD_3OD.

Figure S4. ^{1}H-^{1}H-COSY spectrum of anthenoside V (**1**) in CD_3OD.

Figure S5. HSQC spectrum of anthenoside V (**1**) in CD_3OD.

Figure S6. HMBC spectrum of anthenoside V (**1**) in CD_3OD.

Figure S7. ROESY spectrum of anthenoside V (**1**) in CD_3OD.

Figure S8. HRESIMS spectrum of the mixture of anthenosides W and X (**2** + **3**).

Figure S9. ^{1}H-NMR spectrum of the mixture of anthenosides W and X (**2** + **3**) in CD_3OD.

Figure S10. ^{13}C-NMR spectrum of the mixture of anthenosides W and X (**2** + **3**) in CD_3OD.

Figure S11. ^{1}H-^{1}H-COSY spectrum of the mixture of anthenosides W and X (**2** + **3**) in CD_3OD.

Figure S12. HSQC spectrum of the mixture of anthenosides W and X (**2** + **3**) in CD_3OD.

Figure S13. HMBC spectrum of the mixture of anthenosides W and X (**2** + **3**) in CD_3OD.

Figure S14. ROESY spectrum of the mixture of anthenosides W and X (**2** + **3**) in CD_3OD.

Figure S1. HRESIMS spectrum of anthenoside V (**1**).

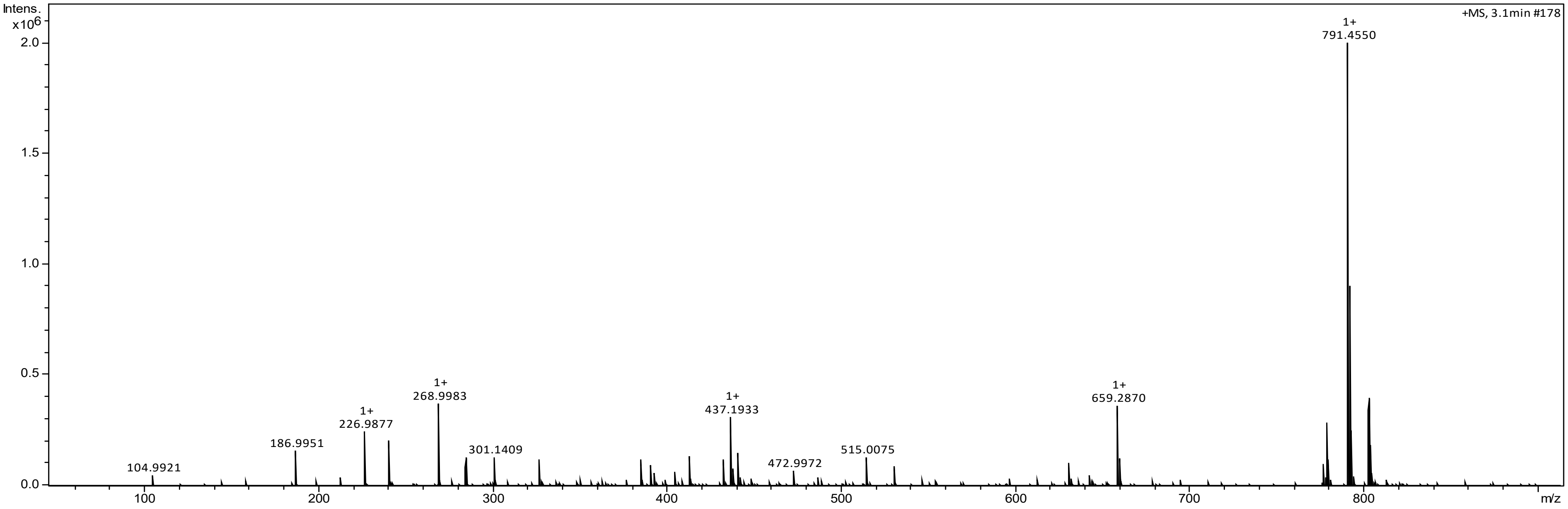

Figure S2. ^{1}H-NMR spectrum of anthenoside V (**1**) in CD_3OD.

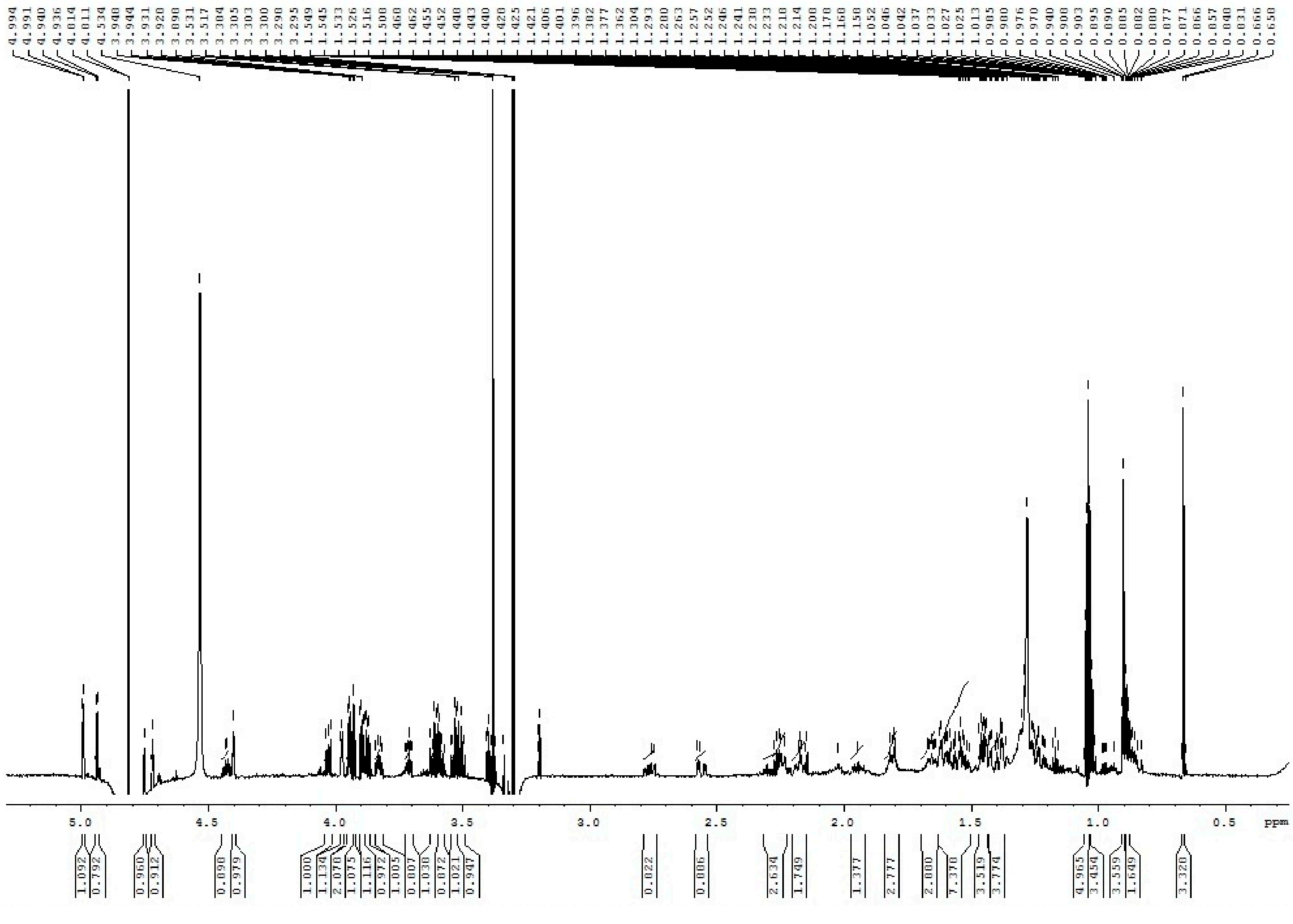

Figure S3. ^{13}C-NMR spectrum of anthenoside V (**1**) in CD_3OD.

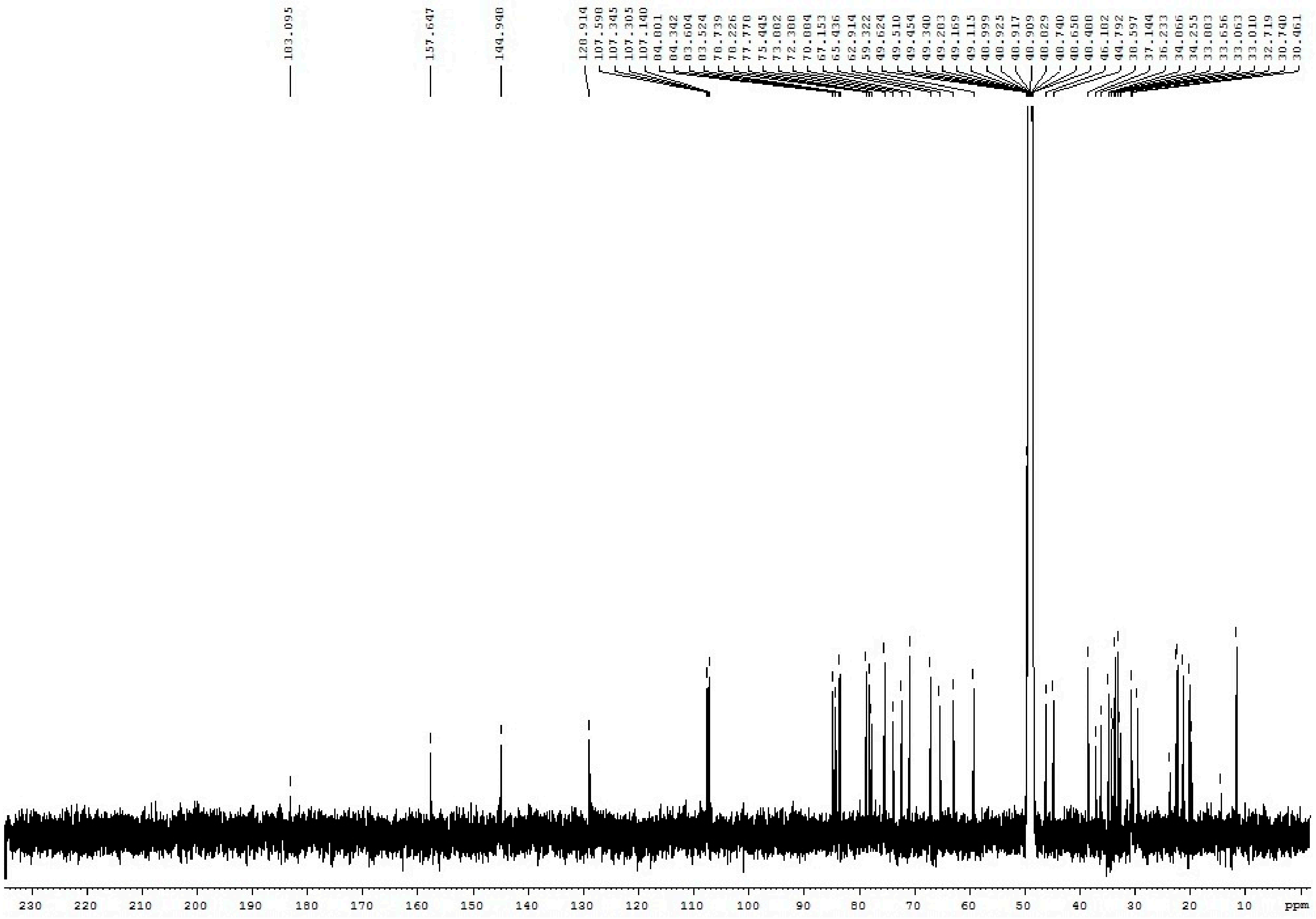

Figure S4. ^1H-^1H-COSY spectrum of anthenoside V (**1**) in CD_3OD.

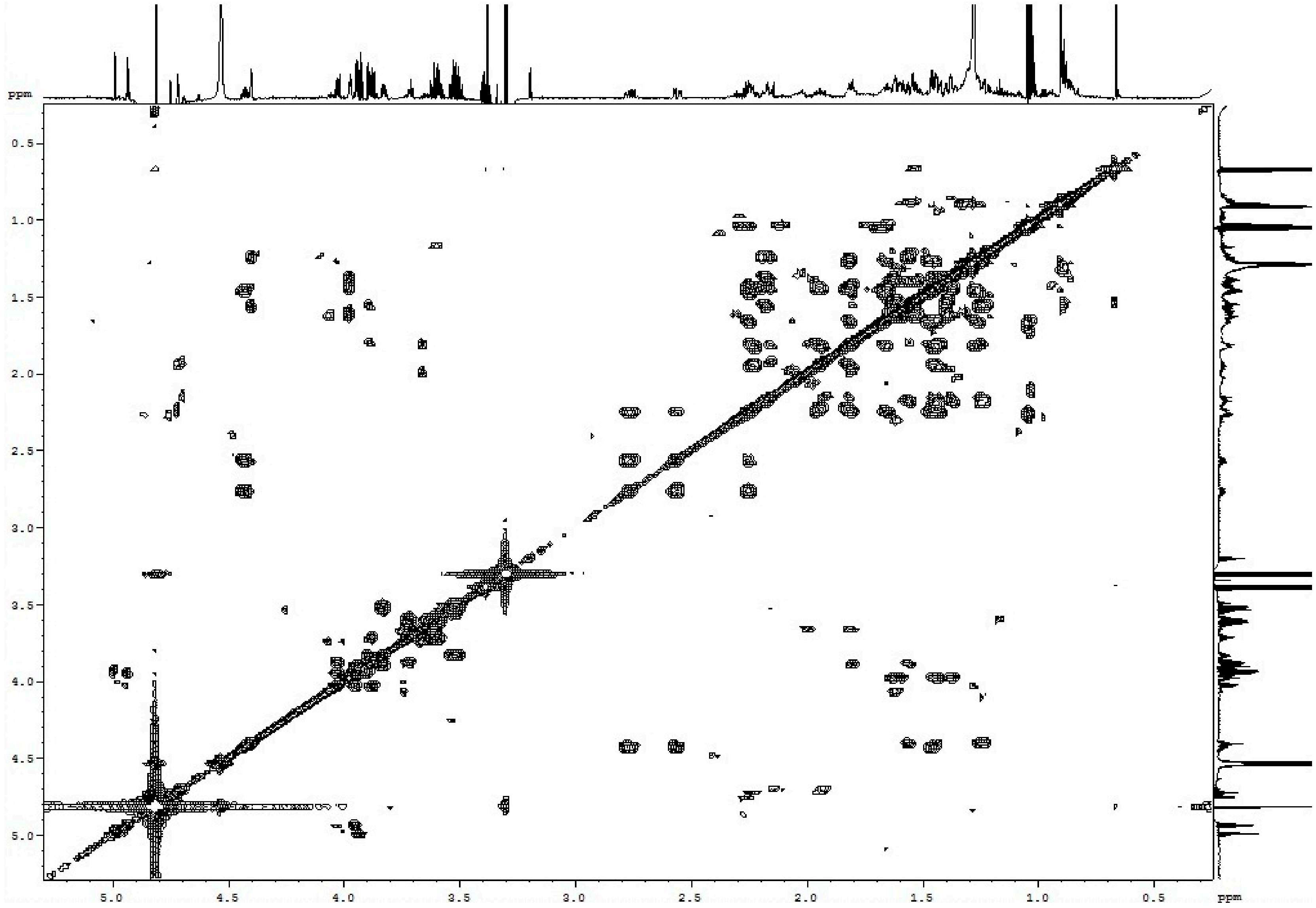

Figure S5. HSQC spectrum of anthenoside V (**1**) in CD_3OD.

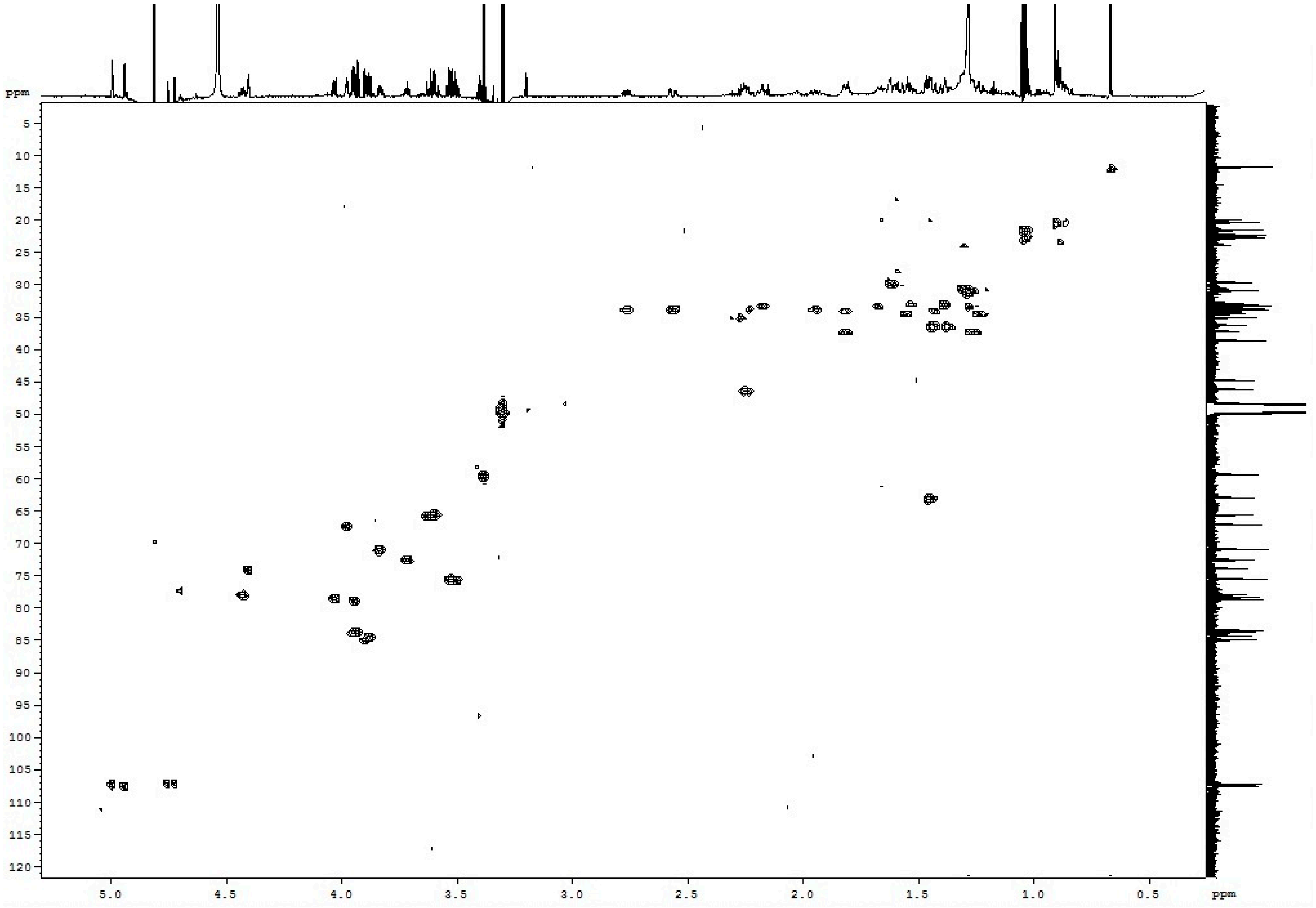

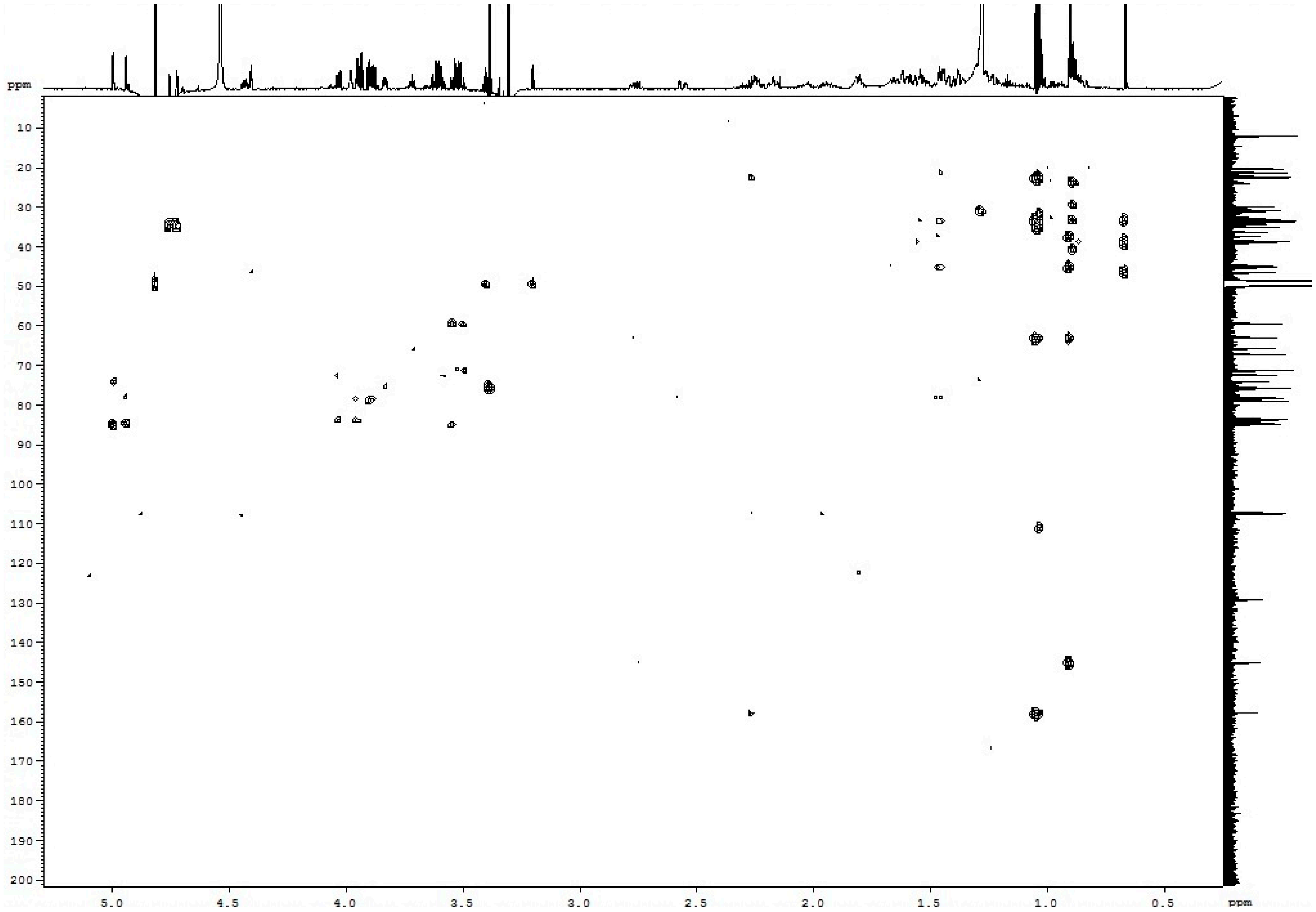

Figure S6. HMBC spectrum of anthenoside V (**1**) in CD_3OD.

Figure S7. ROESY spectrum of anthenoside V (**1**) in CD_3OD.

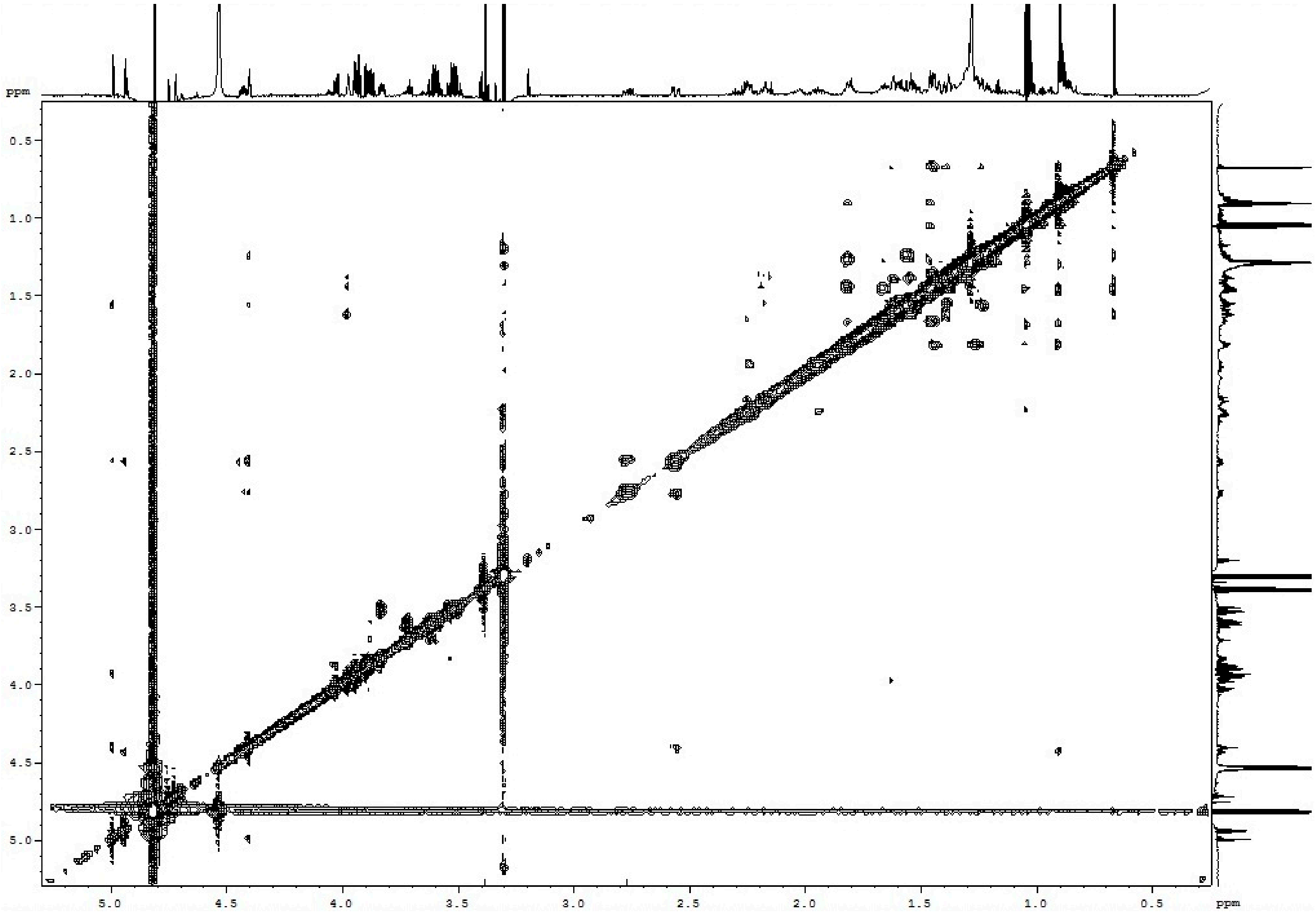

Figure S8. HRESIMS spectrum of the mixture of anthenosides W and X (**2** + **3**).

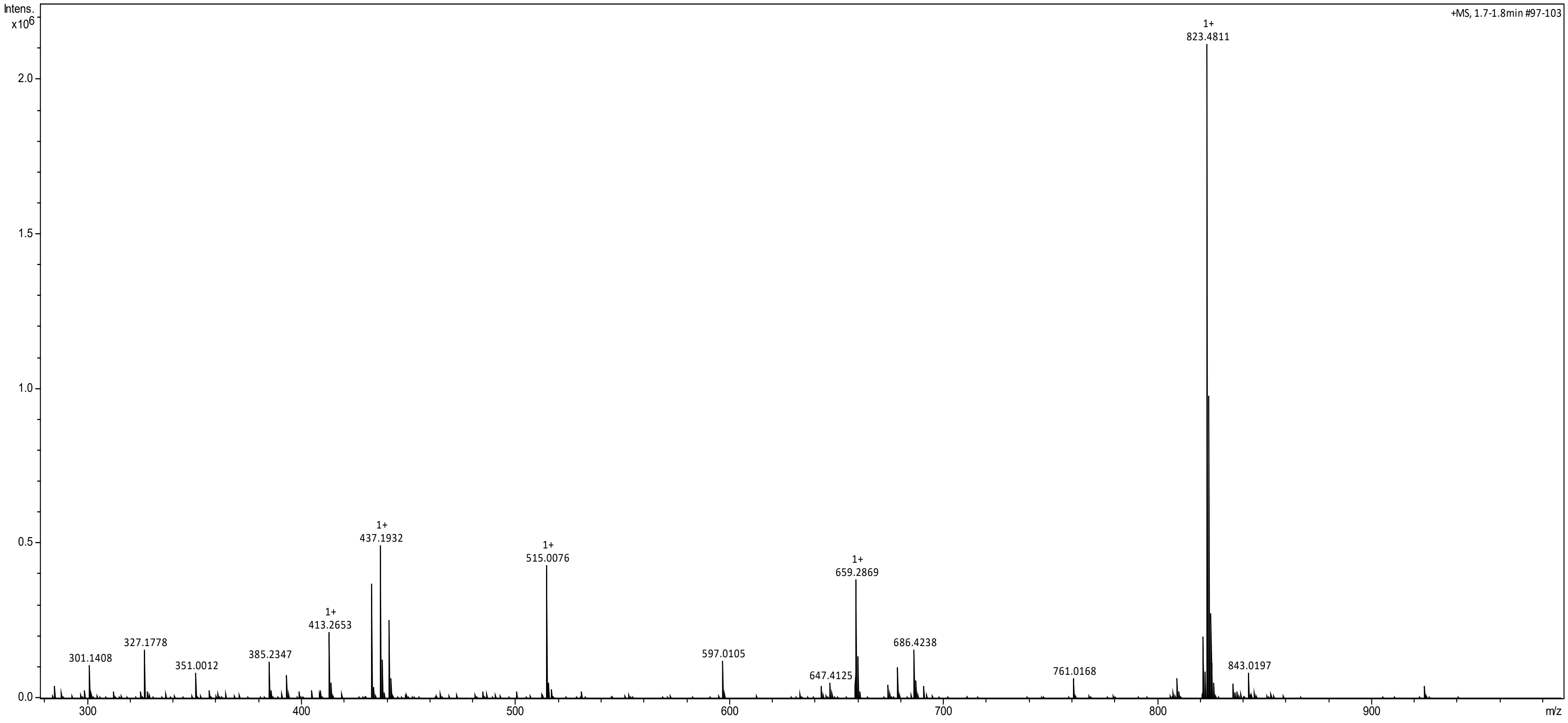

Figure S9. ^{1}H-NMR spectrum of the mixture of anthenosides W and X (**2** + **3**) in CD_3OD.

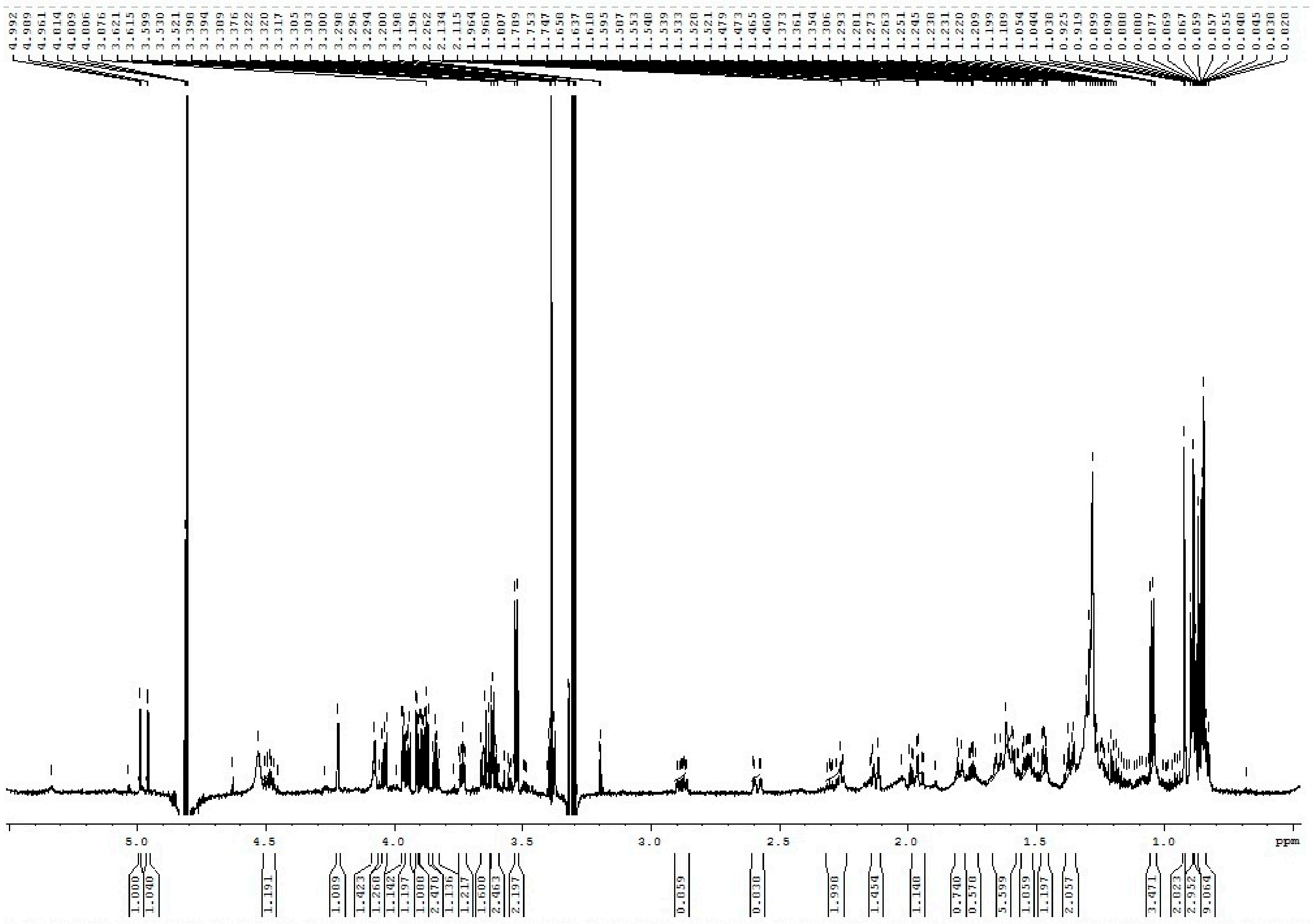

Figure S10. ^{13}C-NMR spectrum of the mixture of anthenosides W and X (**2 + 3**) in CD_3OD.

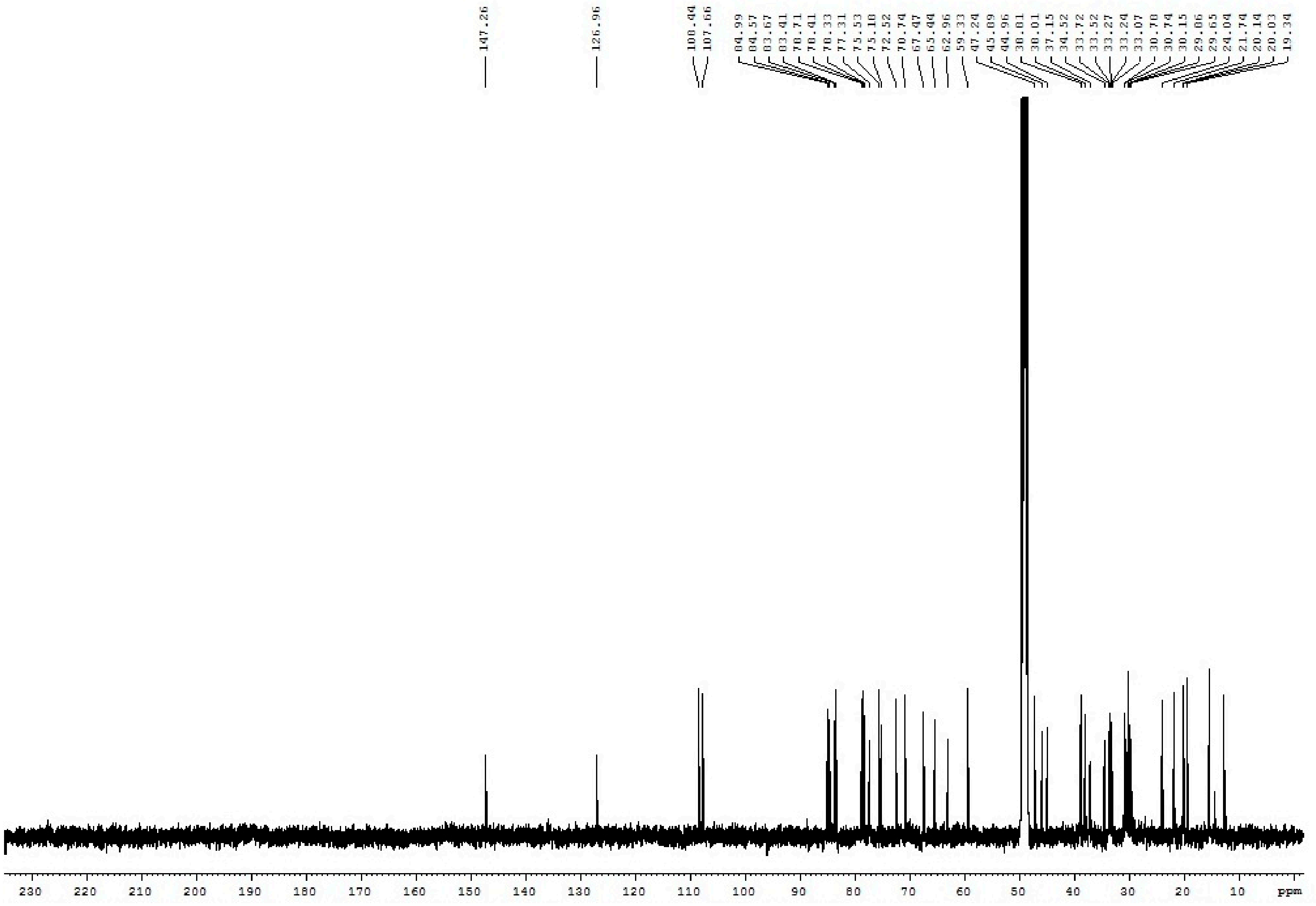

Figure S11. ¹H-¹H-COSY spectrum of the mixture of anthenosides W and X (**2** + **3**) in CD_3OD.

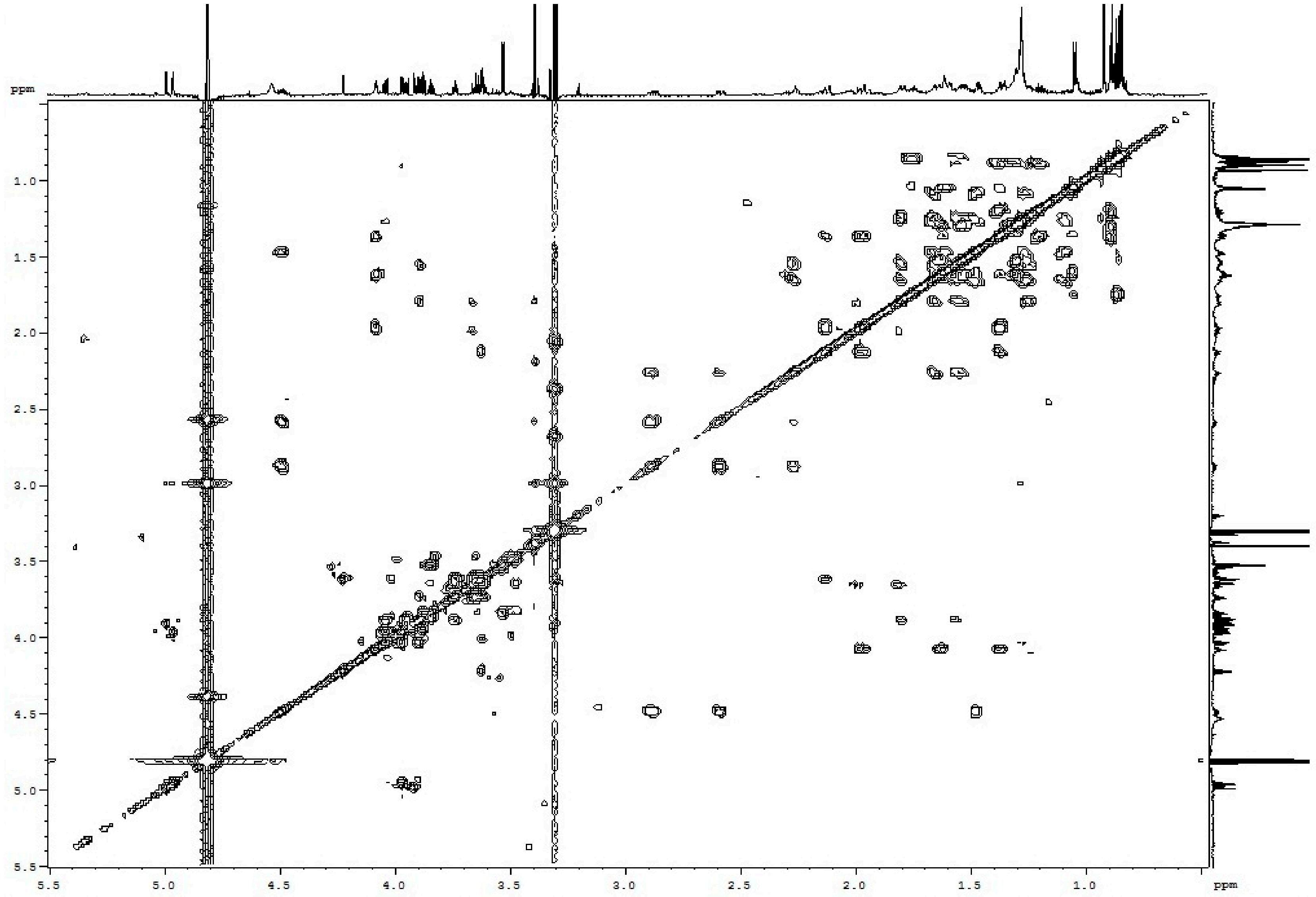

Figure S12. HSQC spectrum of the mixture of anthenosides W and X (**2** + **3**) in CD_3OD.

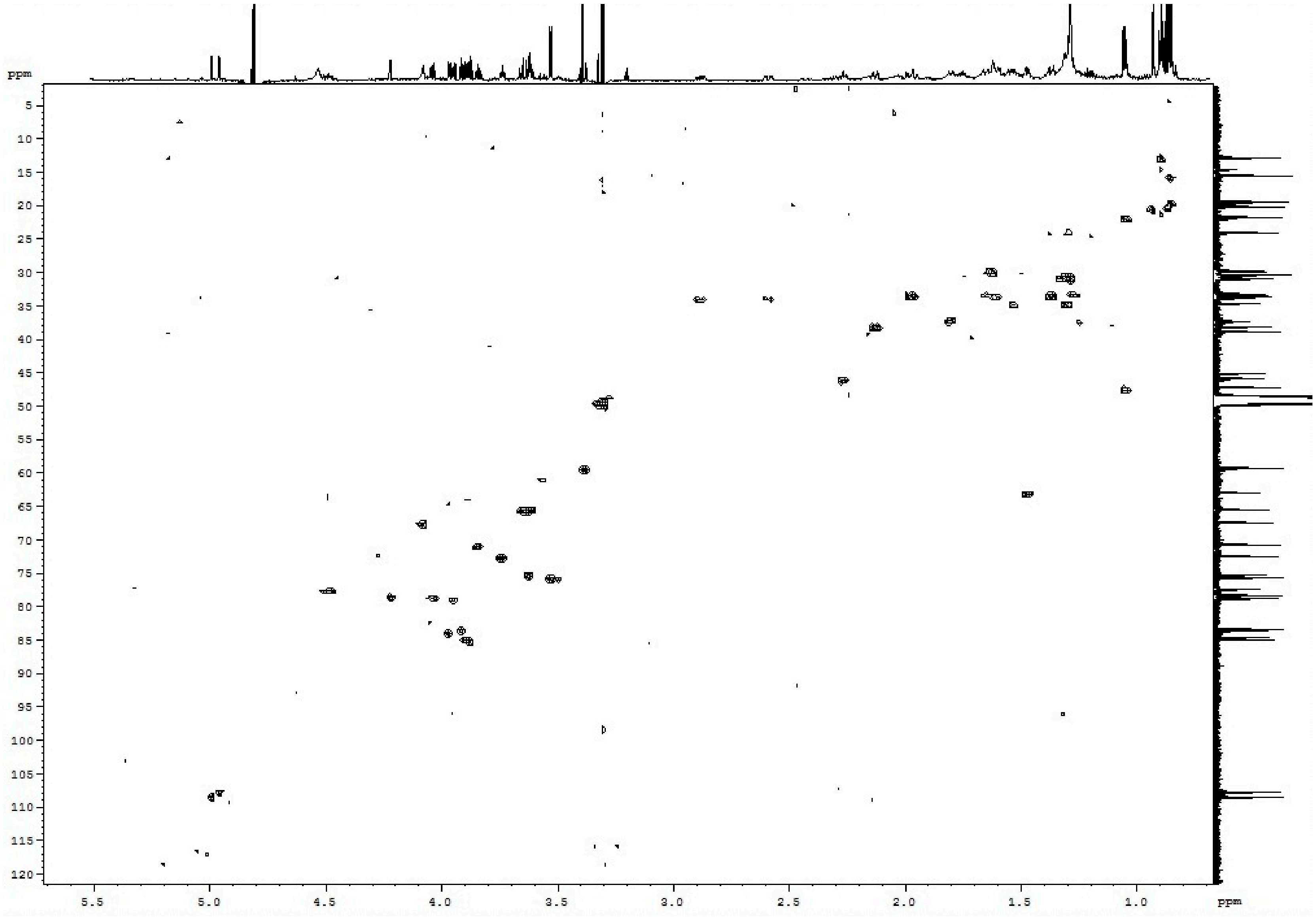

Figure S13. HMBC spectrum of the mixture of anthenosides W and X (**2** + **3**) in CD_3OD.

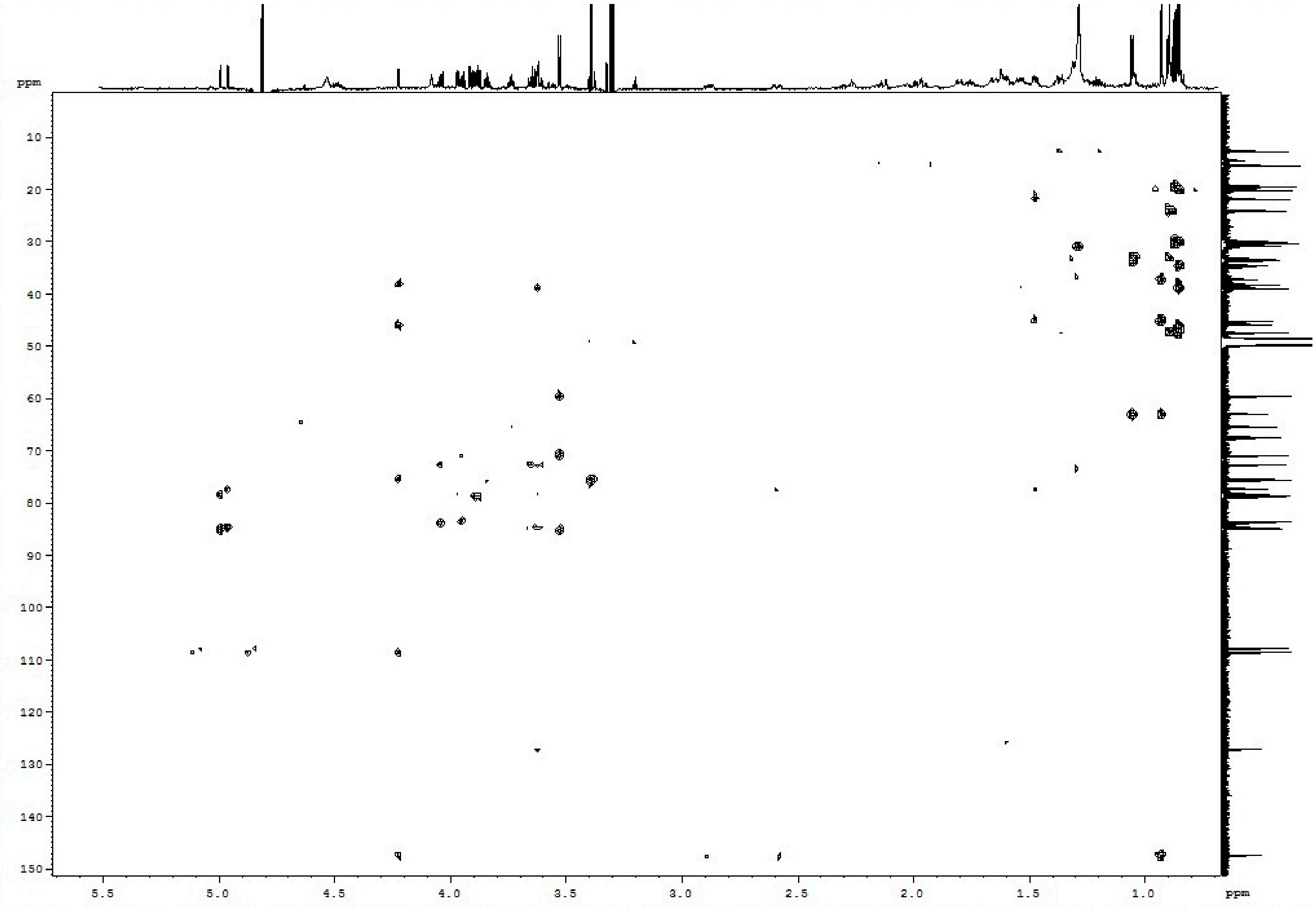

Figure S14. ROESY spectrum of the mixture of anthenosides W and X (**2** + **3**) in CD_3OD.

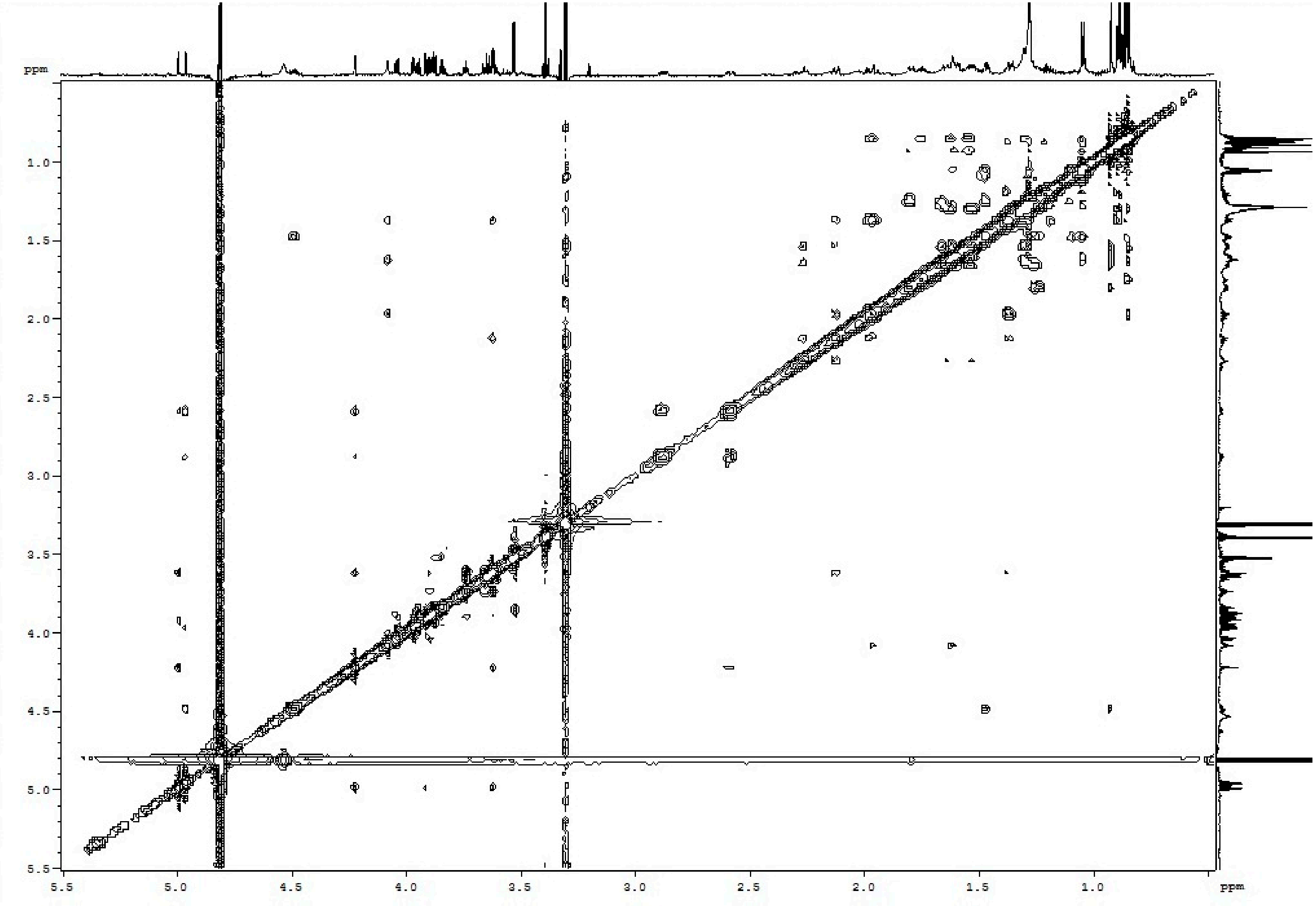

Supplement: Supplementary file 1 [file marinedrugs-16-00420-s001.pdf]
